# Discriminating abiotic and biotic organics in meteorite and terrestrial samples using machine learning on mass spectrometry data

**DOI:** 10.1093/pnasnexus/pgaf334

**Published:** 2025-11-18

**Authors:** Daniel Saeedi, Denise Buckner, Thomas A Walton, José C Aponte, Amirali Aghazadeh

**Affiliations:** School of Electrical and Computer Engineering, Georgia Institute of Technology, Atlanta, GA 30332, USA; Astrochemistry Laboratory, Goddard Space Flight Center, NASA, Greenbelt, MD 20771, USA; School of Electrical and Computer Engineering, Georgia Institute of Technology, Atlanta, GA 30332, USA; Astrochemistry Laboratory, Goddard Space Flight Center, NASA, Greenbelt, MD 20771, USA; School of Electrical and Computer Engineering, Georgia Institute of Technology, Atlanta, GA 30332, USA

**Keywords:** astrobiology, mass spectrometry, machine learning, meteorites

## Abstract

With the upcoming sample return missions to the Solar System where traces of past, extinct, or present life may be found, there is an urgent need to develop unbiased methods that can distinguish molecular distributions of organic compounds synthesized abiotically from those produced biotically but were subsequently altered through diagenetic processes. We conducted untargeted analyses on a collection of meteorite and terrestrial geologic samples using 2D gas chromatography coupled with high-resolution time-of-flight mass spectrometry and compared their soluble nonpolar and semipolar organic species. To deconvolute the resulting large dataset, we developed LifeTracer, a computational framework for processing and downstream machine learning analysis of mass spectrometry data. LifeTracer identified predictive molecular features that distinguish abiotic from biotic origins and enabled a robust classification of meteorites from terrestrial samples based on the composition of their nonpolar soluble organics.

Significance Statement Determining whether organic molecules in planetary samples originate from biological or nonbiological processes is central to the search for life beyond Earth. Yet, distinguishing these origins is challenging due to overlapping chemical signatures and limited access to pristine extraterrestrial materials. We assembled a rare and valuable dataset comprising high-resolution mass spectrometry profiles from meteorites and terrestrial soils and developed LifeTracer, a machine learning framework that identifies subtle patterns distinguishing biotic from abiotic origins. In contrast to traditional biomarker-based approaches, LifeTracer analyzes untargeted chemical signatures to infer molecular origin with high accuracy. It enables scalable, unbiased biosignature detection and offers a powerful tool for interpreting complex organic mixtures returned by current and future planetary missions.

## Introduction

Future sample return missions such as NASA’s Mars Sample Return (MSR) and JAXA’s Martian Moons eXploration (MMX) are set to return samples from the Martian system to Earth, including some from environments that may have harbored life. These missions, crucial to answering questions about the origins of life, demand developing unbiased strategies to assess the dividing line between samples containing abiotically synthesized organic compounds and those containing biotic remnants of past or present life (1–3).

Among relevant analog materials, carbonaceous chondrites are a primitive and organically rich class of meteorites, representing the oldest solid materials in the Solar System available for laboratory analyses. The soluble organic compounds in carbonaceous meteorites constitute a record of abiotic chemical reactions predating the emergence of life as we know it (4–6). In contrast, terrestrial geologic samples such as soils and lake sediments contain preserved organic compounds of biotic origin, which serve as molecular fossils recording the degradation products from living organisms (7, 8). While meteorites and terrestrial materials have been studied independently, and differences in distributions of individual compound classes have been delineated (5, 9, 10), the full inventory of soluble organic compounds between these sample types has not been systematically compared, leaving a gap in our understanding of how the overall molecular distributions of soluble organics differ for abiotic and biotic synthesis/degradation. This gap limits our ability to robustly distinguish abiotic and biotic samples.

Although abiotic and biotic processes can yield similar classes of molecules, their underlying synthetic mechanisms differ, leading to distinct molecular distributions that may indicate their origin. Abiotic organics in meteorites typically arise from ion-radical reactions in cold astrochemical environments (11), whereas biotically synthesized molecules reflect enzymatic processes and cellular functions (9). As a result, meteorites and terrestrial samples can, in some cases, be distinguished by features such as isoprenoid structures or the L-enantiomeric excess observed in the 20 proteinogenic amino acids (5). However, in the absence of such biosignatures, an extraterrestrial sample could be misclassified as purely abiotic despite exhibiting organic patterns inconsistent with abiotic synthesis on Earth. Therefore, reliable classifications should be based on the analysis of the overarching inventory of organic compounds rather than on the absence of specific biosignatures, which may provide only indirect or Earth-biased evidence of present and/or past life.

The challenge of distinguishing abiotic from biotic organic signatures becomes particularly complex when considering the effects of diagenesis, thermal alteration, and contamination that can modify original molecular patterns over geological timescales. Traditional analytical approaches often focus on specific biomarker compounds or isotopic signatures, but these targeted methods may miss the broader molecular context that could provide more robust discrimination criteria. Furthermore, while the inherent complexity of organic mixtures in both meteoritic and terrestrial materials necessitates comprehensive analytical frameworks capable of handling high-dimensional datasets and extracting meaningful chemical patterns, the limited availability of well-characterized extraterrestrial samples constrains the volume of data available and restricts the range of methods that can be effectively applied.

**Fig. 1.**
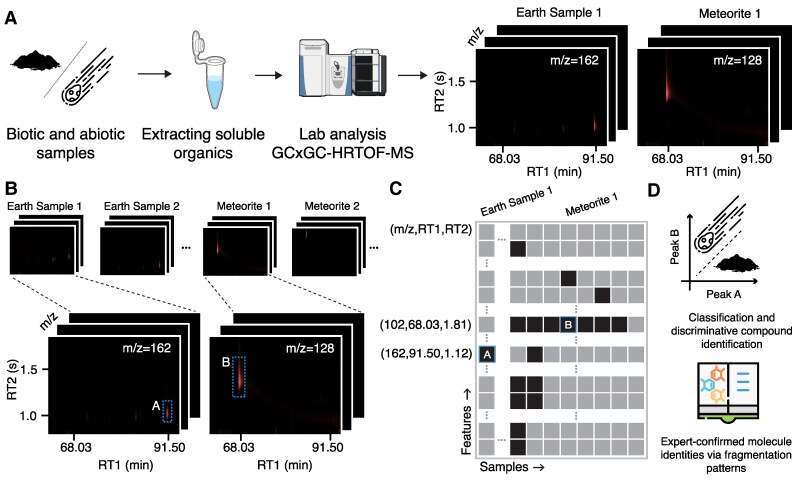
The LifeTracer workflow for collecting, curating, and analyzing the mass spectrometry data and developing a machine learning model for classifying samples. A) The soluble nonpolar and semipolar organics in 8 meteorites and 10 terrestrial geologic samples were analyzed using untargeted 2D gas chromatography coupled to high-resolution time-of-flight mass spectrometry (GC×GC-HRTOF-MS), resulting in total ion images (TIIs) in four dimensions corresponding to the mass-over-charge ratio (m/z), retention time in the first column (RT1), retention time in the second column (RT2), and intensity (abundance). This illustration shows the workflow for Meteorite 1 (Aguas Zarcas) and Earth Sample 1 (Iceland soil), with distinct peaks at m/z=162 and 102 amu, respectively. The device shown as a cartoon schematic to illustrate the instrument layout. B) High-intensity peaks in TIIs are extracted. Peaks may represent fragment ions originating from the same parent compound. C) Peaks are clustered and tabulated with rows representing features and columns representing samples. Black and gray squares indicate the presence or absence of features, respectively. In this illustration, the squares marked as A and B correspond to the peaks at m/z = 162 and 102 amu in Aguas Zarcas and Iceland soil samples. D) A logistic regression model is trained on the processed data to classify samples into the abiotic and biotic classes based on the composition of their organic compounds. Features with large regression coefficients are analyzed to identify the organic compounds that play a key role in distinguishing between biotic and abiotic samples. We manually analyzed the fragmentation patterns and exact masses in comparison to standards to determine the identity or candidate molecule type for each discriminative compound discovered by LifeTracer.

We hypothesized that meteorite samples can be distinguished from terrestrial geologic samples based on differences in the distributions of their organic compounds. To test this hypothesis, we analyzed a suite of meteoritic and terrestrial samples (Table S1) and systematically compared the molecular distribution of their organic contents using 2D gas chromatography coupled to high-resolution time-of-flight mass spectrometry (GC×GC-HRTOF-MS). To interpret the resulting high-dimensional data, we developed a computational framework called LifeTracer to quantify differences between samples of abiotic (meteoritic) and biotic (terrestrial) origin (Fig. 1). Leveraging analyte metrics from GC×GC-HRTOF-MS, LifeTracer extracts molecular abundances, distributional patterns, and mass fragmentation spectra (m/z) of soluble nonpolar and semipolar organic compounds. A logistic regression model trained on compound-level features achieved over 87% accuracy in classifying unseen samples as meteoritic or terrestrial (Table S2 and Fig. S1). Crucially, LifeTracer identified an unbiased set of discriminative features across sample types, enabling classification based on organic distributions, without relying on the presence or absence of known biosignatures.

**Fig. 2.**
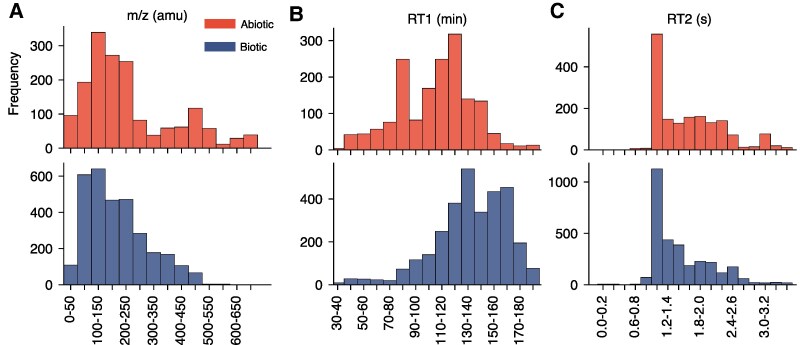
Comparison of mass-to-charge ratio (m/z), first retention time (RT1), and second retention time (RT2) distributions between meteoritic (abiotic, top) and terrestrial (biotic, bottom) fragment ions. A) Mass-to-charge ratio (m/z, in amu) distribution showing different molecular weight patterns. B) First retention time (RT1, in minutes) distribution revealing significantly earlier elution of meteoritic compounds ($P<10^{-208}$), reflecting the higher volatility of compounds in samples with abiotic origins. C) Second retention time (RT2, in seconds) distribution. All three parameters showed statistically significant distributional differences between abiotic and biotic samples ($P<10^{-20}$, $P<10^{-203}$, and $P<10^{-21}$, respectively).

**Fig. 3.**
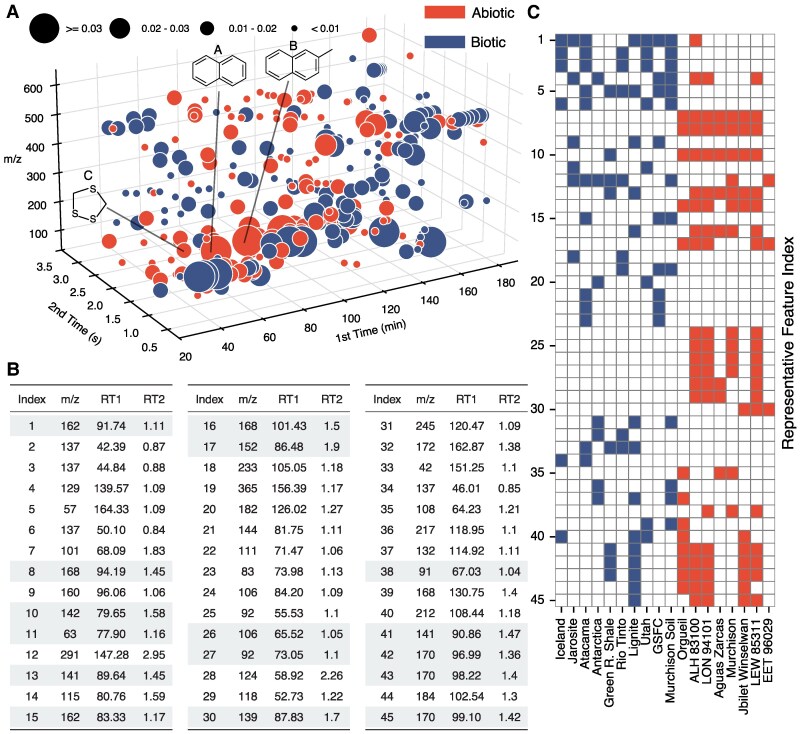
Visualization of the distribution of compounds in meteoritic samples and terrestrial geologic samples and the regression coefficients of the logistic regression model trained in LifeTracer. A) Observing that fragment ions (features with similar RT1 and RT2 but varying m/z) consistently ranked among the top features (see Figs. S4C, D and S6C, D), we grouped features within 50 s in RT1 and 0.8 s in RT2, defining these as feature groups. The feature with the highest absolute coefficient was selected as the representative feature in each feature group. The scatter plot comprises an inventory of unbiased predictive feature groups in our logistic regression model. The size of the spheres represents the magnitude of the regression coefficients of the representative feature (importance in classification), and the color indicates the direction of enrichment: red for abiotic and blue for biotic samples. Naphthalene (labeled as A, index = 7) and 2-methylnaphthalene (labeled as B, index = 10) were highlighted as notable predictive features. Among the top 45 feature groups, 1,2,4-trithiolane (labeled C, index = 28), containing sulfur heteroatoms alongside carbon and hydrogen, emerged as a predictive feature group for abiotic samples. B) A table presents all the feature groups whose corresponding representative features are shared across at least two samples. These feature groups are sorted by regression coefficient. Gray rows indicate polycyclic aromatic hydrocarbons (PAHs) with attached alkyl groups such as methyl, ethyl, or propyl. This panel shows the top 45 feature groups after excluding those attributed to spectral artifacts or internal instrument contaminants; the complete list is provided in Table S5. C) Heatmap depicting representative features corresponding to the table in B) across different samples. Columns represent different samples, and rows correspond to the representative feature indices White cells indicate the absence of a feature in a sample, while red (abiotic) and blue (biotic) cells denote the presence of the feature in the respective sample.

By adopting a holistic view of sample composition, LifeTracer maximizes information extraction from each dataset, including low-abundance, unidentified, or otherwise nondiagnostic compounds (Figs. 2 and 3). Our approach offers a significant improvement over traditional targeted methods, which rely on manual compound identification and often neglect the broader chemical context. Furthermore, by identifying and aligning recurring feature groups across samples, LifeTracer also functions as a discovery tool, highlighting analytes of potential interest for downstream investigation.

## Results

We analyzed eight carbonaceous meteorites (Murchison, Orgueil, ALH 83100, LON 94101, LEW 85311, EET 96029, Aguas Zarcas, and Jbilet Winselwan) and ten terrestrial geologic samples (lignite, Green River Shale, and soils from Antarctica, Atacama, Rio Tinto, Jarosite, the Murchison fall site, Utah, Goddard Space Flight Center [GSFC], and Iceland). This collection represents samples across two distinct pools: extraterrestrial specimens containing vestiges of abiotic organic synthesis and terrestrial samples containing trace organics from past and/or present life (Table S1).

We conducted an untargeted analysis of the soluble organic species across nonpolar and semipolar compound classes (Fig. 1A). GC×GC-HRTOF-MS measured the mass-to-charge ratio (m/z), the retention time in the first dimension (RT1), the retention time in the second dimension (RT2), and the intensity of compounds in the samples. Total ion images (TIIs) were constructed by aggregating signal intensities across m/z values from 30 to 700 amu, RT1 values from 2,200 to 11,458 s (sampled every 3.5 s), and RT2 values from 0 to 3.5 s (sampled every 8 ms), resulting in images with a resolution of 2,643×439 pixels (Fig. 1A, S2, and S3). Peaks corresponding to fragment ions of highly abundant compounds were extracted following peak alignment and clustering (Methods, Fig. 1B, Fig. S4A and B). A total of 9,475 peaks were identified across the meteorite samples and 9,070 across the terrestrial samples (Fig. S5A and B and Table S3). On average, meteorite samples contained 1,184 peaks and terrestrial samples 907, where each peak represents a unique fragment ion rather than a full analyte.

We observed statistically significant differences in the distributions of m/z, RT1, and RT2 values between meteoritic and terrestrial samples by Kolmogorov–Smirnov test ($P<10^{-20}$, $P<10^{-203}$, and $P<10^{-21}$, respectively; Fig. 2A–C). Notably, the RT1 values of fragment ions in meteorite samples were significantly lower than those in terrestrial samples by Mann–Whitney *U* test ($P<10^{-208}$), consistent with higher compound volatility in abiotic materials, as more volatile species elute earlier during GC thermal ramps (see Fig. 2B and Methods section). A comprehensive visualization of the processed data and interactive plots are available online^a^.

To prepare the data for downstream machine learning, we clustered chromatographic peaks with identical m/z and retention times within 50 s in RT1 and 0.8 s in RT2. Each resulting cluster, referred to as a feature, corresponds to a parent compound or fragment ion characterized by a unique (m/z, RT1, RT2) triplet (Figs. S4A, B and S6A, B). This resulted in 9,152 features (rows), each detected in at least one sample. We trained a logistic regression model on this processed dataset, which accurately classified unseen samples with over 87% test accuracy and an AUC greater than 0.93 (Tables S2 and S4, Figs. S1 and S7). Analysis of the regression coefficients revealed that the top predictive features often corresponded to fragment ions derived from the same parent compound (Figs. S4C, D and S6C, D). These ions exhibited similar RT1 and RT2 values but varied in m/z, suggesting coelution and common origin. Based on this observation, we grouped features that appeared within 50 s in RT1 and 0.8 s in RT2, regardless of m/z, into broader units termed feature groups. Within each group, the feature with the largest absolute regression coefficient was designated as the representative. This procedure yielded 140 feature groups, each shared across two or more samples and potentially corresponding to distinct analytes (Table S5).

After identifying feature groups and completing the classification of abiotic and biotic samples, we manually analyzed their fragmentation patterns and exact masses by comparison to analytical standards and published databases to determine the identities or candidate molecular classes of the representative feature in each feature group. Of the 140 feature groups whose representative feature was shared across at least two samples, 31 were matched to known organic analytes, 17 corresponded to unidentified compounds with tentative class assignments, and 23 were unidentified. The remaining 69 feature groups were attributed to spectral artifacts or internal instrument contaminants, such as siloxanes originating from the GC analysis. To assess the impact of noise, we zeroed the regression coefficients associated with artifact-related feature groups and found that the classification accuracy of the logistic regression model remained unchanged, confirming that model performance was not dependent on these spurious features (Fig. S1). For each authentic analyte, we quantified relative abundance using peak area as reported by the LECO ChromaTOF software. As a validation step, we cross-referenced each sample in which LifeTracer identified a given feature group with its actual presence in the raw data. Fourteen of the 31 authentic compounds were confirmed to appear exclusively in the samples flagged by LifeTracer. The remaining 17 compounds were also present in additional samples not initially flagged, though typically in lower abundance, with minor retention time shifts or coeluting with background ions. The identified authentic compounds included polycyclic aromatic hydrocarbons (PAHs) and their alkylated homologs, polycyclic isoprenoids, environmental contaminants, and oxygen-, chlorine-, or sulfur-containing organics.

A polysubstituted C6-alkylbenzene with the formula ${\text{C}}_{12}{\text{H}}_{18}$ emerged as the most predictive feature group for biotic samples, found in Iceland, Jarosite, Atacama, Utah, GSFC, and Murchison Soil, while it was also present in ALH 83100. Possibly 9-methyldecalin or a structural isomer appeared as the second most predictive feature group for biotic samples, found in Atacama, Rio Tinto, Utah, and Murchison Soil (representative feature index = 2 in Fig. 3). Naphthalene emerged as the most predictive feature group for abiotic samples, identified by LifeTracer in every meteorite except EET 96029 (labeled A; representative feature index = 7 in Fig. 3). A possible methyl biphenyl or another isomer with the formula ${\text{C}}_{13}{\text{H}}_{2}$ appeared as the second most predictive feature group for abiotic samples and was detected in all abiotic samples except EET 96029 (labeled B; representative feature index = 10 in Fig. 3). Notably, among the top 45 feature groups, 1,2,4-trithiolane (labeled C; index = 28 in Fig. 3A) stands out as the predictive feature group for abiotic samples containing heteroatoms (sulfur) in addition to carbon and hydrogen. Another prominent observation is the frequent occurrence of alkylated PAHs, that is, PAHs with attached alkyl groups such as methyl, ethyl, or propyl, which appeared 16 times (highlighted in gray in Fig. 3B), compared to only a single occurrence of a PAH (index = 7 in Fig. 3B and C) among the top 45 feature groups. Their exact structures have not been elucidated, but manual analysis of fragmentation patterns and comparison of exact masses to the National Institute of Standards and Technology (NIST) database indicates that these fragment groups correspond to the aforementioned compounds or families. Manual identification of all feature group molecules was achieved through the comparison of sample data to known mass fragmentation patterns and/or matched to retention times of standards.

## Discussion

### Organics as molecular targets in the search for life beyond Earth

Previous targeted analyses of organics in meteorites and on Earth have demonstrated distinct differences in their distributions. These patterns can help differentiate whether organics in a terrestrial sample of unknown origin are likely to have formed through abiotic or biotic mechanisms. Meteorites contain short and highly branched carboxylic acids and acyclic hydrocarbons (10), hundreds of often racemic amino acids (5), PAHs but no isoprenoids (12), and a variety of nucleobases (13). On the other hand, life synthesizes carboxylic acids and acyclic hydrocarbons with long carbon chains with specific and nonrandom branches to form cell membranes (10), a library of 20 amino acids with a preference for the L-enantiomer that builds proteins (5), multiringed polycyclic triterpenoids composed of linked isoprene units for cell signaling and structural reinforcement (8), and five nucleobases for constructing information-coding RNA and DNA (13).

However, if an extraterrestrial sample from a potentially habitable environment lacks classical biosignatures such as molecular asymmetry (e.g. isoprenoid-derived chiral fatty acids and sterols), high organizational complexity (e.g. RNA, DNA), or enantiomeric excess (L-amino acids or D-sugars), could it still represent an alternate form of life? Carbon-based alien biology should be expected to follow similar biochemical principles to life on Earth, surviving by producing organic molecules that fulfill cellular functions through directed synthesis pathways. The resulting inventory would, in turn, differ from organics formed through random abiotic reactions, and this deviation in molecular distributions from an abiotic norm can also serve as a potential fingerprint of extraterrestrial life.

Our study directly addressed this challenge by analyzing the distributional properties of organic compounds, which can originate from both abiotic and biotic synthetic pathways. Meteorite and terrestrial samples each contain tens of thousands of organic molecules, making it analytically difficult to resolve and identify low-abundance isomers with similar masses and polarities (14). As a result, many compound classes remain understudied, and systematic comparisons of their global distributional patterns in abiotic versus biotic contexts have been lacking.

We bridged this gap by applying 2D GC×GC-HRTOF-MS to achieve fine-grained separation of individual isomers in natural samples, followed by machine learning to quantitatively compare their distributional patterns. This approach enables us to move beyond the search for specific biomarkers and toward a broader, data-driven framework for distinguishing between abiotic and biotic chemical signatures.

### Distinct differences in the distribution of PAHs across meteoritic and terrestrial geologic samples

While PAHs have been reported in previous studies of meteoritic samples (15–19), their appearance as top predictive compounds at the dividing line between abiotic meteoritic and biotic terrestrial samples is an important outcome of this study (Table S5). PAHs emerged as the top predictive compounds in our analysis. The differences observed in their structures and distributions reflect the known distinctions between biotic and abiotic synthesis mechanisms. In meteorites, ion-radical processing in cold environments builds PAHs through the cyclization of short aliphatic chains and the addition of more rings through the fluoranthene and pyrene pathways (20). On Earth, PAHs represent breakdown products formed through burning fuel, where starting materials (i.e. coal and oil) are sourced from diagenetically altered biomass. Through these processes, incomplete combustion can produce PAHs with various numbers of rings and alkyl branching, depending on the composition of the starting material and the extent of combustion (21, 22). Additional aromatic molecules found in terrestrial samples include polycyclic triterpenoids, multiringed molecules with aromatic constituents composed of linked isoprene units (8). Differences in the sources and synthesis pathways responsible for the formation of these molecules are reflected in the differences that we enumerate in their distributions across biotic and abiotic natural samples (Table S5).

### Relevance to the search for life beyond Earth

Ascertaining whether organics of unknown origin were synthesized by abiotic or biotic processes is a challenging but critical task in astrobiology. Although samples of biotic origin on Earth typically contain numerous biomolecules that are diagnostic of life and not natively found in abiotic meteoritic samples, a host of additional less-specific organics are also present in both types of samples. Consequently, while many individual molecules in extraterrestrial and terrestrial samples may be nondiagnostic of either biotic or abiotic chemistry, differences in the overall distributions of organics can indicate their origin and serve as a whole as potential biosignatures that can be exploited in the search for life beyond Earth. It is reasonable to expect that on other planets, organic molecules of ambiguous origin will likely be present within a mixed signal of organic carbon synthesized by numerous different processes, including exogenous infall, in situ abiotic synthesis, and potentially life and their corresponding degradation products. Indeed, numerous organics have already been detected in Martian materials, including both Martian meteorites fallen to Earth and samples analyzed in situ by the Curiosity and Perseverance rovers. Considering the complex mixture that should be present in these extraterrestrial samples, a thorough and multivariate approach like LifeTracer will be needed to analyze overall organic content to assess potential similarities or differences to known terrestrial and meteoritic samples of abiotic and biotic origin, especially in the light of Martian sample return missions. Machine learning coupled with traditional manual data analysis can provide a powerful pathway towards accomplishing this goal.

## Methods

### Materials and reagents

All glassware used for sample extraction was pyrolyzed in a muffle furnace at 500 ^∘^C overnight to remove residual organics. Terrestrial and meteorite sample masses are listed in Table 1 below. Each meteorite and terrestrial geologic sample was crushed to a fine powder in an individual mortar and pestle, transferred to a 5 mL borosilicate glass vial, and extracted using 300 ^∘^C for 24 h. Extracts were separated from mineral residues by centrifugation and removed to a total recovery vial (1 mL). One microliter aliquots of each extract was analyzed via direct injection on a GC×GC-HRTOF-MS (LECO HRT+ 4D system). Standards and reagents were purchased from Sigma-Aldrich and Thermo Scientific and used without further purification.

**Table 1.** Meteoritic abiotic and terrestrial biotic samples and masses used for this study.

Meteoritic samples		Terrestrial samples	
ALH 83100	201.32	Antarctica soil	204.57
Aguas Zarcas	200.03	Atacama Desert soil	200.94
EET 96029	200.86	Green River Shale	200.11
Jbilet Winselwan	202.39	GSFC soil	200.09
LEW 85311	200.45	Iceland soil	201.07
LON 94101	201.86	Jarosite Panoche valley	203.63
Murchison	203.05	Lignite	200.75
Orgueil	200.11	Murchison fall site soil	201.40
		Rio Tinto springs soil	200.47
		Utah desert soil	200.43

A detailed description of samples is outlined in Table S1. The unit for the numbers is mg.

### Analysis of solvent-soluble organic compounds

Samples of the DCM extracts and procedural blanks were analyzed without derivatization using an Agilent 7890B gas chromatograph coupled to a LECO Pegasus HRT+ 4D time-of-flight mass spectrometer operated in full-scan mode over the m/z range of 50–500 (ion source set at 250 ^∘^C and 60 eV). The primary GC oven was equipped with a 5 m base-deactivated fused silica guard column (Restek, 0.25 mm I.D.) and two Rxi-5 ms (30 m length × 0.25 mm I.D. × 0.5^∘^C at 1 ^∘^C/min, held for 5 min at 60 ^∘^C, then increased to 110 ^∘^C at 2 ^∘^C/min, held for 5 min at 110 ^∘^C, then increased to 260 ^∘^C at 2 ^∘^C/min, held for 5 min at 260 ^∘^C, finally increased to 280 ^∘^C at 20 ^∘^C/min, and held for 25 min at 280 ^∘^C. The secondary oven offset temperature was kept at 5 ^∘^C relative to the primary oven, the modulation temperature offset was kept at 15 ^∘^C, and a modulation period of 5 s was applied. The carrier gas used was ultrahigh purity grade helium (5.0 grade) at 1.4 mL/min. Duplicate injections of samples were made in the splitless mode in aliquots of 2μL. Data were processed using the LECO Corp. ChromaTOF software. Mass spectra were used to identify compounds compared to reference standards where possible. The detection limit was estimated to be >0.5 pmol of analyte/μL injected, while the quantitation limits were ∼1 pmol of analyte/μL injected.

### GC × GC-HRTOF-MS

2D GC×GC-HRTOF-MS is a powerful tool for performing untargeted analysis to separate, identify, and quantify a variety of soluble organic compounds within complex mixtures. While traditional GC-MS uses a single chromatographic column to separate analytes by volatility and interactions with the stationary phase; eluting compounds are sent to the mass spectrometer where they are ionized, fragmented, and identified based on their mass-to-charge (m/z) ratio, GC×GC-HRTOF-MS employs a second column controlled by a secondary oven, where cuts exiting the first column are flash cooled and then thermally ramped again through the second column, achieving a higher degree of separation. GC plots analytes as a 1D chromatogram, but compounds with the same m/z values often co-elute, precluding identification in complex mixtures like extraterrestrial extracts. On the other hand, the second column and oven in a GC×GC-HRTOF-MS enable two separations to plot analytes in 2D space. When applied to natural samples, GC×GC-HRTOF-MS typically results in a 5-fold increase in sensitivity and a 3-fold increase in the number of molecules identified compared to traditional GC (23).

### GC × GC-HRTOF-MS data format

The raw data from the GC×GC-HRTOF-MS device comprise seven columns for each scan (i.e. rows of the table). The seven columns in the raw data are labeled as row index, spectrum, 1st time (s), 2nd time (s), TOF, m/z, area, and resolution. The row index labels each scan, ranging from 0 to 535,018,068. The spectrum ranges from 0 to 1,157,499. First time (s) is the retention time in the first column in seconds (denoted by RT1) ranging from 2,200 to 11,457.568 s. Second time (s) is the retention time in the second column in seconds (denoted by RT2) ranging from 0 to 3.504 s. TOF denotes the time of flight ranging from 117,226.668 to 478,162.121 s. m/z is the mass-over-charge ratio (denoted by m/z) ranging from 29.99778 to 700.02157 amu. Area is the intensity of the scan (denoted by I) ranging from 11.41 to 2,416,640.00, and resolution indicates ranges from 3,520 to 2,097,120. To analyze the GC×GC-HRTOF-MS data and extract peaks in this study, we focused on m/z, RT1, RT2, and I.

### Analyzing the GC × GC-HRTOF-MS data

The analysis of the GC×GC-HRTOF-MS data comprises five steps: (i) data quantization, (ii) ion image creation, (iii) peak detection, (iv) peak filtering, and (v) peak clustering. Herein, we describe these five steps in detail:

- Data quantization. We quantized the scan entries based on their m/z values, ranging from 30 to 700, with increments of 1. For each m/z value *v*, we searched the data within each sample, collected scans with the same RT1 and RT2 values and m/z in the range [v−0.5,v+0.5], and grouped them into a single entry by aggregating their Area (Fig. S2). We used the pivot_table function from the Pandas library in Python, setting the columns parameter to RT1, the index parameter to RT2, and the values parameter to Area. This resulted in a table where rows correspond to the RT2 and columns correspond to the RT1. Missing intensities were filled with 0 (zero imputation). We created fixed-size tables corresponding to RT1 ranging from 2,200 to 11,457.568 s and RT2 from 0 to 3.504 s, respectively. We imputed the columns and rows missing RT1 and RT2 values with zero (see Fig. S3).
- Ion image creation. The tables from the previous step comprise a collection of 2D heatmaps for each m/z in each sample. These heatmaps, called the total ion images (TIIs), collect the total intensity value for every (RT1, RT2) pair. We visualized all the TIIs as part of our GC×GC-HRTOF-MS preprocessing pipeline and provided them as Supplementary material.
- Peak detection. For each TII, we first found the standard deviation of intensity value denoted as *σ*. We considered the pixels with intensity values larger than $\lambda_{1}\sigma$, where $\lambda_{1}$ is a constant. We set the rest of the values with intensity lower than $\lambda_{1}\sigma$ to zero (23). We input the resulting TII into a clustering algorithm called Density-Based Spatial Clustering for Applications with Noise (DBSCAN). DBSCAN (24) clusters the isolated pixels in TIIs into groups and outputs the coordinates of the rectangular region that encompasses those isolated pixels (Figs. S4B and S6B). In DBSCAN, we set the parameters min_sample=20 and eps=5, which allowed us to remove isolated pixels. Using the automatic parameter calibration outlined in (vi), we selected $\lambda_{1}=5$. If a cluster’s width and height exceed this 50 and 1 s, respectively, the algorithm will split it recursively along the RT1 and RT2 dimension.
- Peak filtering. Due to the untargeted nature of our analysis, we expected noisy peaks in GC×GC-HRTOF-MS. We developed a rigorous filtering strategy to remove the noisiest peaks while retaining the valid ones. We followed a conservative denoising procedure, including four consecutive filters applied to the extracted peaks: (i) Overall TII Filtering. We identified TIIs with high-intensity pixels uniformly distributed across the image as noise (Fig. S8A) and thus discarded TII with more than 10% nonzero pixels. (ii) Local total intensity filtering. We defined the total intensity of a peak as the summation of intensities within each rectangular region ($I_{\mathrm{rect}}$). We discarded the peaks with $I_{\mathrm{rect}}<\lambda_{2}\sigma$ (Fig. S8B). Following the automatic parameter calibration described in (vi), we set $\lambda_{2}=100$. 3) Filtering Noisy Regions. We considered vertical and horizontal strip regions on the TII as noise (Fig. S8C and D). To address this issue, we visually inspected 10% of all TIIs, focusing on regions where vertical and horizontal noise appeared. We observed that these regions had intensities larger than half the maximum intensity. Consequently, we set intensity values lower than $\lambda_{1}\sigma$ to zero. Next, we performed a ratio test, comparing the number of nonzero pixels to the total number of pixels. If the ratio of nonzero pixels exceeded the specific thresholds identified in Table S6, we discarded any peaks within these regions. This approach ensured that our filtering was neither too strict, which might risk losing relevant peaks, nor too lenient, which could allow noise to pass through.
- Peak clustering. In GC×GC-HRTOF-MS, misalignments cause larger shifts along the RT2 axis and smaller shifts along the RT1 axis (see Figs. S4B and S6B). We grouped peaks within a maximum distance of ${\text{RT1}}_{\mathrm{thrsh}}$ for RT1 and ${\text{RT2}}_{\mathrm{thrsh}}$ for RT2, while having the same m/z, and considered them a single feature for downstream machine learning analysis (see Fig. 1C and Figs. S4B and S6B). The presence of these features is denoted by a value of 1, while their absence is denoted by a value of 0 in Fig. 1C. Based on automatic parameter calibration described in (vi), we selected ${\text{RT1}}_{\mathrm{thrsh}}=50$ s, and ${\text{RT2}}_{\mathrm{thrsh}}=0.8$ s to strike a balance: a larger ${\text{RT1}}_{\mathrm{thrsh}}$ might cause a feature to represent multiple compounds instead of one while still allowing for some variation in the RT1 values of the same compound.
- Parameter calibration. We developed an automatic calibration procedure using a small, expert-verified reference set of six compounds across eight meteorite samples with known presence/absence patterns (Fig. S9A). Through grid search over $\lambda_{1}\in \{1,\ldots ,20\}$ and $\lambda_{2}\in \{1,10,20,\ldots ,200\}$, we evaluated each parameter pair by running peak detection and measuring accuracy, defined as successful recovery of reference compounds within tolerance windows of ±50 s in RT1 and ±1 s in RT2. Rather than selecting parameters yielding maximum accuracy (which risks admitting excessive spurious peaks and creating impractical downstream analysis burdens due to an overwhelmingly large number of feature groups), we adopted a conservative approach: among configurations achieving greater than 90% accuracy, we selected the largest $\lambda_{1}$ to maximize noise rejection, then chose the median $\lambda_{2}$ for that $\lambda_{1}$ to avoid being either too restrictive or too permissive with peak detection. This yielded $\lambda_{1}^{*}=5$ and $\lambda_{2}^{*}=100$ (Fig. S9B). For clustering thresholds, we calculated retention time dispersions for each reference compound as the difference between maximum and minimum RT1, and similarly for RT2, across all samples where that compound was detected (Fig. S9C and D). Using maximum dispersions ensures our clustering accommodates worst-case retention time variability. The final thresholds were RT1 threshold of 50 s (rounded from 49.056 s to the nearest second) and RT2 threshold of 0.8 s (rounded from 0.816 s to 0.1 s) (Fig. S9D).

### Training the abiotic against the biotic classifier

We conducted a comprehensive 9-fold cross-validation for model selection, with nested leave-one-out cross-validation within each fold. To achieve this, we randomly shuffled the 18 samples and split them into nine groups (i.e. folds) of two samples. We used eight folds to train the model and the last to test it. To set the hyperparameters of the classifier within each fold, we performed a leave-one-out procedure: we left one sample out of the training set for validation. We trained the model on the remaining 15 samples (Fig. S10). This procedure was repeated for all 15 samples and the hyperparameters that yielded the highest average classification accuracy on the validation samples were selected. We then used these hyperparameters to find the test accuracy of the model on the unseen fold. We repeated these procedures for all nine folds and considered the classification accuracy across the nine folds as test accuracy. We repeated the entire procedure ten times with ten random shuffles of the processed data to avoid bias toward any specific split and averaged the test accuracies across the ten seeds.

In addition to the previous evaluation approach, we implemented a nested stratified K-fold cross-validation strategy to provide a more systematic assessment of model performance across different classifier models. Our validation framework consisted of two nested loops. The outer loop used a 6-fold stratified split to divide samples into training and test sets while ensuring at least one sample from each class appeared in every fold. For each of the six outer folds, one fold was held out as the test set, while the remaining five folds were used for hyperparameter optimization. The hyperparameter selection process worked as follows: Using the five remaining folds, we performed 5-fold cross-validation where each fold served as a validation set while the other four folds were used for training. For each combination of model hyperparameters, we calculated AUC scores across all five validation folds and selected the hyperparameters that achieved the highest average validation AUC. We then evaluated the model with these optimal parameters on the held-out test fold. This process was repeated for all six outer folds, and we averaged the AUC scores across all six test folds to obtain the final performance metric. To ensure robust results, we repeated this entire nested cross-validation procedure across ten different random seeds. The comprehensive results from this analysis are presented in Table S4. However, the leave-one-out nested approach described above remains our preferred evaluation method due to the limited sample size (18 samples), where stratified K-fold may create folds with insufficient representation of minority classes, potentially leading to unreliable performance estimates in small-sample classification scenarios.

We considered four models as candidate classifiers for abiotic and biotic samples: Logistic regression (with L1 and L2 norm regularization), support vector machine (SVM), random forest (RF), and naive Bayes classifier. For the logistic regression model, we used the LogisticRegression package within the linear model of sklearn (25) in Python. We used the parameter penalty=l1 for L1 norm regularization and penalty=l2 for L2 norm regularization. We used the default values for all the parameters and only tuned the inverse of regularization strength (*C*). This parameter strikes a balance between the strength of regularization, which helps prevent overfitting, and data fidelity, which ensures the model accurately fits the training data. We considered the values in $\left\{10^{-4},10^{-3},\ldots ,1,10,\ldots ,10^{4}\right\}$ for *C* during hyperparameter tuning, covering a wide range of regularization strengths. For the SVM model, we used the SVC package from the support vector machine algorithms in sklearn. We used the default values for all parameters and only tuned the inverse of regularization strength (*C*) and the kernel type. During hyperparameter tuning, we considered values in $\left\{10^{-3},10^{-2},\ldots ,1,10,\ldots ,10^{3}\right\}$ for *C* and {linear, polynomial,radial basis function,sigmoid} as kernel types.

For the Bernoulli naive Bayes classifier, we used the BernoulliNB package from the naive Bayes algorithms in sklearn. We used the default values for all parameters and only tuned the smoothing parameter *α*. The hyperparameter *α* prevents zero probabilities in the calculations by adding a small constant to the frequency counts of each feature. During hyperparameter tuning, we considered values in {0.01,0.1,0.5,1,5,10} for *α*. For the random forest classifier, we used the RandomForestClassifier package from the ensemble algorithms in sklearn in Python. We used the default values for all parameters and only tuned the number of trees. During hyperparameter tuning, we considered values in {20,50,100,200,500} for the number of trees. For all these models, we used ${\text{RT1}}_{\mathrm{thrsh}}=50\;\text{s}$, ${\text{RT2}}_{\mathrm{thrsh}}=0.8\;\text{s}$, $\lambda_{1}=5$, and $\lambda_{2}=100$. We did not involve $\lambda_{1}$, $\lambda_{2}$, ${\text{RT1}}_{\mathrm{thrsh}}$, and ${\text{RT2}}_{\mathrm{thrsh}}$ in the hyperparameter search because this would have increased the chance of accepting noisy peaks at the expense of higher validation accuracy. We instead fixed the values of $\lambda_{1}$, $\lambda_{2}$, ${\text{RT1}}_{\mathrm{thrsh}}$, and ${\text{RT2}}_{\mathrm{thrsh}}$ before conducting evaluation and parameter selection. Among all the models, logistic regression with L2 norm regularization achieved the highest classification accuracy (Table S2). For the model’s final deployment in the software, we performed leave-one-out cross-validation using all the processed data. This resulted in the final hyperparameters: C=0.1, $\lambda_{1}=5$, $\lambda_{2}=100$, ${\text{RT1}}_{\mathrm{thrsh}}=50\;\text{s}$, and ${\text{RT2}}_{\mathrm{thrsh}}=0.8\;\text{s}$.

### Statistical analysis

We used the Kolmogorov–Smirnov test and Mann–Whitney *U* test for the analysis of the distribution of values m/z, RT1, and R2 in Fig. 2A–C. The number of peaks for abiotic and biotic samples was 9,475 and 9,070, respectively, which represent the sample sizes used in this analysis. The Kolmogorov–Smirnov test, which is more sensitive to differences in the shape of distributions, showed significant differences for all three parameters. For m/z, the test statistic was 0.146 with $P<10^{-20}$. For RT1, the test statistic was 0.456 with $P<10^{-203}$, and for RT2, the test statistic was 0.148 with $P<10^{-21}$. In all three cases, we rejected the null hypothesis, indicating that the distributions of m/z, RT1, and RT2 for abiotic and biotic peaks are significantly different. The results of the Mann–Whitney *U* test indicated that the m/z distribution of abiotic peaks was not significantly lower than that of biotic peaks (P>0.99), leading us to fail to reject the null hypothesis. Similarly, for RT2, the *P*-value was extremely high (P>0.99), and we also failed to reject the null hypothesis, indicating that abiotic peaks do not have RT2 values significantly higher than biotic peaks. However, for RT1, the *P*-value was extremely low ($P<10^{-208}$), leading us to reject the null hypothesis. This elucidates that the abiotic peak distribution for RT1 is significantly lower than the biotic peak distribution.

### Defining organic feature groups

Analyzing the regression coefficients of the regression model (Figs. S4C and S6C), we found that the top predictive features were fragment ions from the same parent compound. These ions shared similar RT1 and RT2 values but had different mass-to-charge ratios (m/z). Observing the fragment ions consistently appearing among the top features, we grouped these features (referred to as feature groups) using the following procedure. First, we sorted the features in descending order based on the absolute value of their regression coefficients (Figs. S4C and S6C). Starting with the feature that had the highest absolute coefficient, we identified all features within a maximum distance of 50 s in RT1 and 0.8 in RT2, with varying m/z values, grouped them into a single group, and removed them from the ranked features list. We selected the feature with the highest absolute coefficient in each group as the representative feature. We repeated this process for subsequent groups until no features remained. We ranked each group based on the regression coefficient of its representative feature (Figs. S4D and S6D).

### Compound identification with manual analysis of GC × GC-HRTOF-MS data

Data were processed using the LECO Corp. ChromaTOF software. Analytes were manually identified by comparison to authentic standards, using GC×GC-HRTOF-MS retention times, fragmentation patterns, and the accurate mass of parent ions. When pure standards were unavailable, assignments of compounds or families were made based on comparisons of accurate masses and fragmentation patterns with library spectra (15). Relative abundances are reported as peak areas of each analyte, but absolute quantification of analytes was not conducted.

## Supplementary Material

pgaf334_Supplementary_Data

## Data Availability

All data generated or analyzed in this study have been made publicly available. The Meteorites dataset (https://huggingface.co/datasets/DS-20202/Meteorites_LifeTracer) (doi:10.57967/hf/6408) and the Soil Samples dataset (https://huggingface.co/datasets/DS-20202/SoilSample-LifeTracer) (doi:10.57967/hf/6409) can be accessed online. The processed data (https://huggingface.co/datasets/DS-20202/LifeTracer-Processed-Data) are also provided (doi:10.57967/hf/6410). All analysis code is available at GitHub (https://github.com/amirgroup-codes/LifeTracer). The exact version underlying this article is archived at Zenodo (doi:10.5281/zenodo.17050812) and released under the MIT License. The project website provides interactive access to the results and enables online classification of samples (https://life-tracer.github.io/).

## Note

a
https://life-tracer.github.io/

## Previous Presentation

A preliminary version of the work was presented at 56th Lunar and Planetary Science Conference (LPSC) 2025.
