# Supplementary material for: Discriminating abiotic and biotic organics in meteorite and terrestrial samples using machine learning on mass spectrometry data
Source: PNAS Nexus. 2025 Nov 18;4(11):pgaf334. doi: 10.1093/pnasnexus/pgaf334 (PMC12624505; doi:10.1093/pnasnexus/pgaf334)
Supplement: pgaf334_Supplementary_Data [file pgaf334_supplementary_data.pdf]

SI Appendix

Discriminating Abiotic and Biotic Organics in Meteorite and Terrestrial Samples Using Machine Learning on Mass Spectrometry Data

Daniel Saeedi*, Denise Buckner*, Thomas A. Walton, José C. Aponte, and Amirali Aghazadeh

José C. Aponte
E-mail: jose.c.aponte@nasa.gov
Amirali Aghazadeh
E-mail: amiralia@gatech.edu
***D.S. and D.B. contributed equally to this work**

This PDF file includes:

Figs. S1 to S10
Tables S1 to S6
SI References

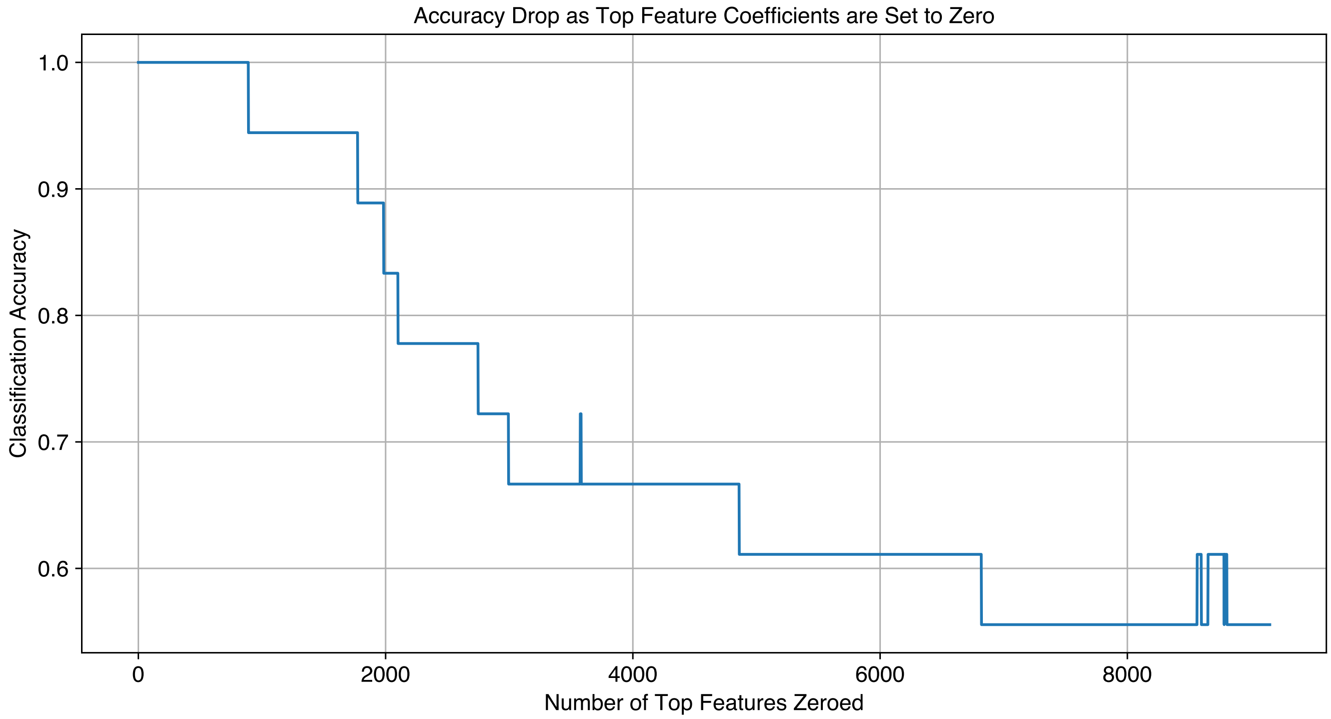

Fig. S1. Impact of feature elimination on classification accuracy. This graph illustrates the decrease in classification accuracy as the coefficients of top-ranked features are progressively set to zero. The x-axis represents the number of top features whose coefficients have been zeroed, while the y-axis shows the resulting classification accuracy. To assess the robustness of our model, we progressively removed top-ranked features and measured classification accuracy. The classification accuracy remained above 90% and 80% when we removed the top 1773 and 2099 features, respectively, demonstrating that LT-Reg relies on a broad range of features rather than a small subset of dominant ones.

a Raw Data

	Spectrum	1st Time (s)	2nd Time (s)	TOF	M/Z	Area	Resolution
0	0	2200.00	0.000	117248.168	30.00884	376.00	10720
1	0	2200.00	0.000	117313.340	30.04225	49.44	11552
2	0	2200.00	0.000	119162.816	30.99811	2952.00	20704
3	0	2200.00	0.000	121051.137	31.98950	5944.00	17760
4	0	2200.00	0.000	121114.773	32.02318	76.88	15968
...	...	...	...	...	...	...	...
203196043	1044999	10557.04	2.952	439384.430	422.17132	24.34	35712
203196044	1044999	10557.04	2.952	441359.938	425.97731	52.38	25728
203196045	1044999	10557.04	2.952	442966.852	429.08576	19.44	33120
203196046	1044999	10557.04	2.952	460617.766	463.97393	142.00	29440
203196047	1044999	10557.04	2.952	461114.430	464.97534	28.03	33920

203196048 rows × 7 columns

b Select scans with an M/Z value between 29.5 and 30.5

	Spectrum	1st Time (s)	2nd Time (s)	TOF	M/Z	Area	Resolution
0	0	2200.00	0.000	117248.168	30.00884	376.00	10720
1	0	2200.00	0.000	117313.340	30.04225	49.44	11552
131	1	2200.00	0.008	117248.145	30.00883	393.50	10048
132	1	2200.00	0.008	117313.531	30.04235	66.88	11232
273	2	2200.00	0.016	117248.105	30.00881	403.50	9536
...	...	...	...	...	...	...	...
203195408	1044998	10557.04	2.944	117299.484	30.03514	27.22	12384
203195409	1044998	10557.04	2.944	117313.004	30.04208	301.00	12576
203195726	1044999	10557.04	2.952	117248.004	30.00875	1486.00	13920
203195727	1044999	10557.04	2.952	117299.438	30.03512	28.00	12256
203195728	1044999	10557.04	2.952	117312.969	30.04206	312.50	12800

1865545 rows × 7 columns

c Group by RT1 and RT2, summing the Area values.

1st Time (s)	2200.000	2203.504	2207.008	2210.512	2214.016	2217.520	2221.024	2224.528
2nd Time (s)								
3.504	102271.42	106637.70	0.00	0.00	105051.55	102278.88	101687.46	0.00
3.496	102237.43	106799.72	107582.72	108742.19	105012.88	102918.65	101594.57	100131.03
3.488	102651.42	107070.01	106995.87	109545.74	105229.35	103347.12	101618.88	100341.08
3.480	103162.35	107171.23	106686.08	110064.11	105307.38	103820.25	101865.96	100325.58
3.472	103730.19	107385.10	106697.96	110503.52	105841.87	103487.22	101655.62	100540.82
...	...	...	...	...	...	...	...	...
0.032	104669.31	102888.89	105243.32	106362.68	109526.52	105334.45	103046.47	101950.18
0.024	104892.07	102676.31	105952.83	106931.52	108375.64	105230.00	103077.03	102053.54
0.016	104942.87	102554.14	105799.61	107396.68	108862.55	105158.24	102500.17	102084.86
0.008	104674.12	102435.63	106207.96	107994.98	108845.42	105008.51	102420.69	101913.36
0.000	105023.60	0.00	0.00	107919.08	108790.73	0.00	0.00	0.00

439 rows × 2386 columns

Fig. S2. Workflow for curating a Total Ion Image (TII) for m/z=30 amu. a, Raw data comprising multiple scans, each represented as a row with associated parameters such as spectrum, retention times (RT1,RT2), TOF, m/z, Area, and Resolution. **b**, Data was filtered to include only scans with m/z values within the range 29.5–30.5, narrowing the dataset to relevant entries. **c**, The filtered data was then aggregated by RT1 and RT2, with Area values summed to produce a final 2D array of dimensions 2386 × 439. Missing (RT1, RT2) pairs were imputed with zero, and the resulting 2D array has dimensions 2643 × 439 for all TIIs across samples.

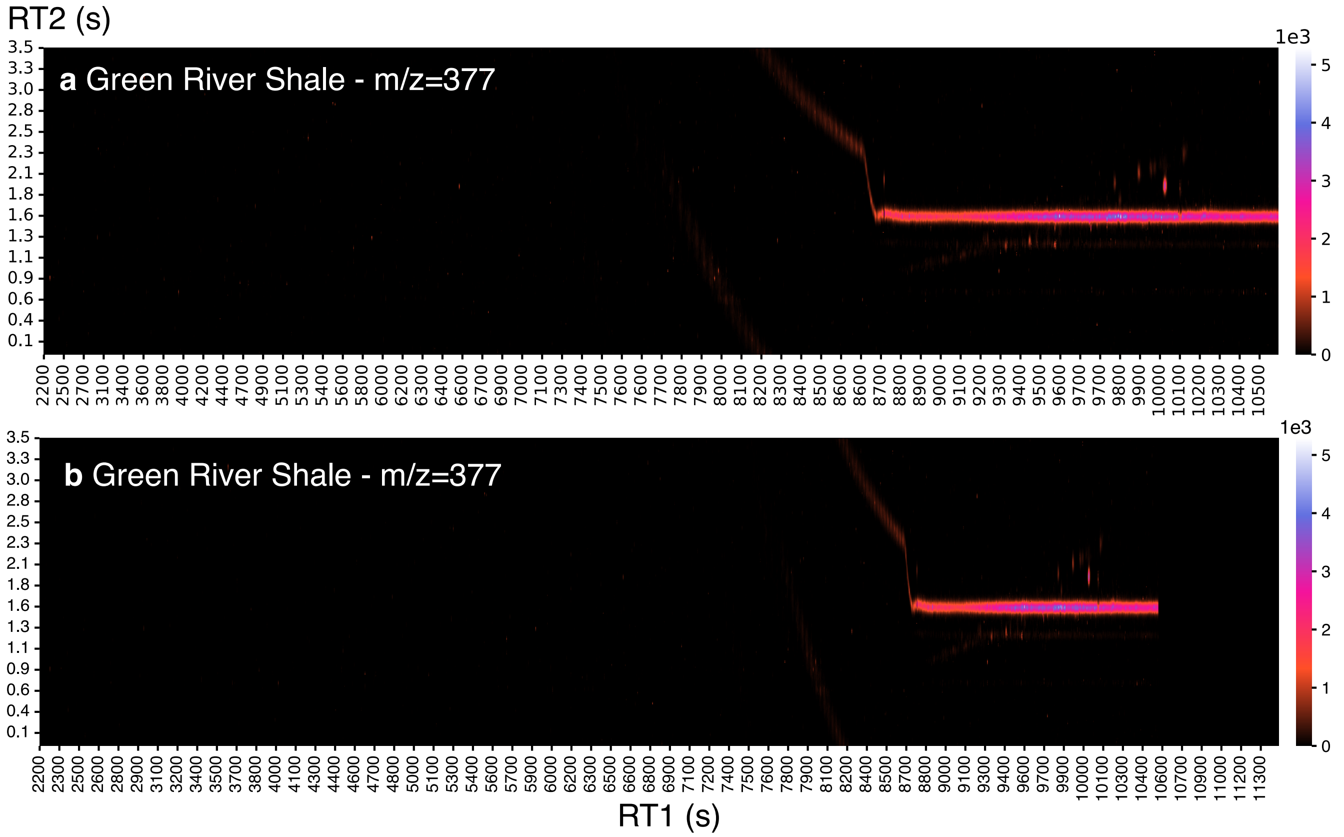

Fig. S3. Data curation pipeline. a, TII displaying missing values for certain RT1 and RT2 intervals, resulting in incomplete data representation. **b**, To standardize the TII across samples and m/z values, the minimum and maximum values of RT1 and RT2 were determined from all TIIs, and a uniform grid was generated with time steps of 3.504 s for RT1 and 8 ms for RT2. Columns or rows corresponding to missing RT1 or RT2 values were filled with zeros, ensuring a complete and consistent TII representation across all samples and m/z.

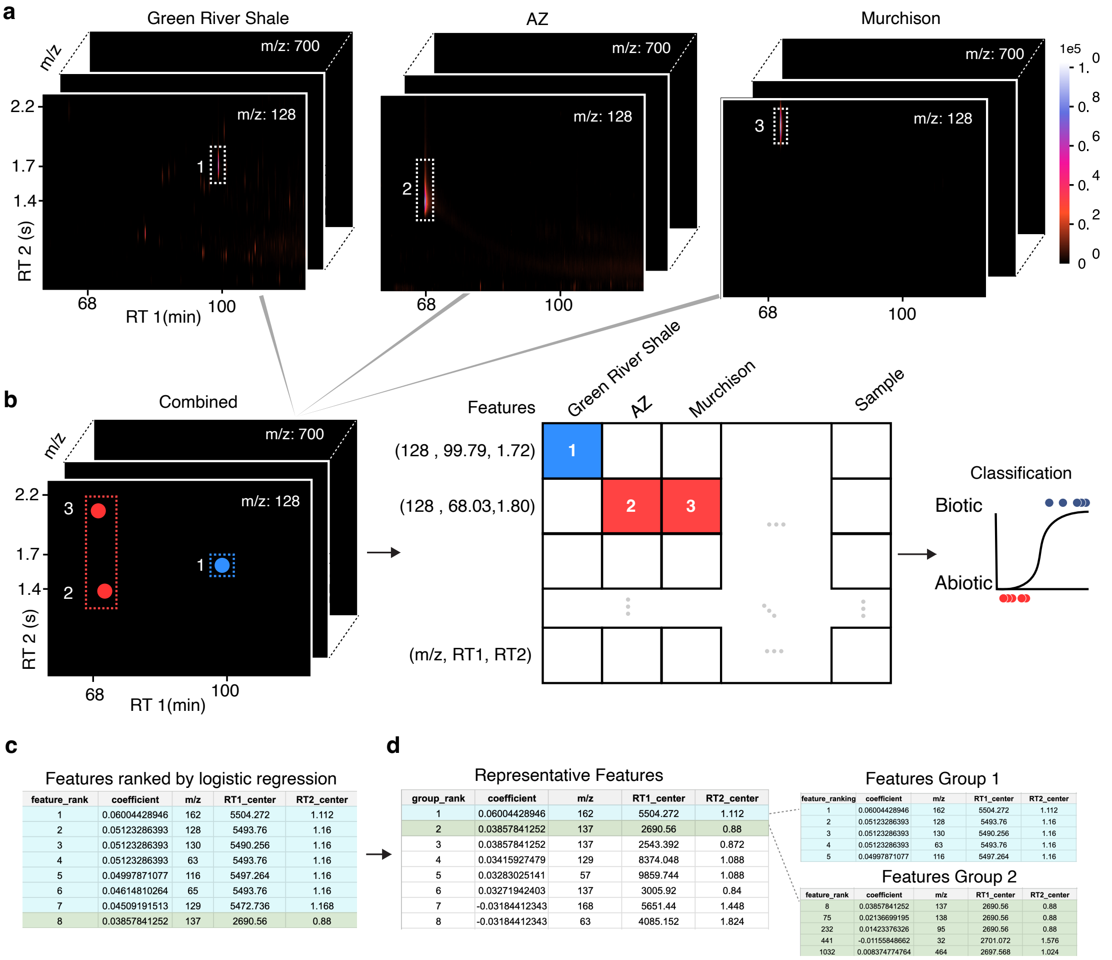

Fig. S4. Schematic of LifeTracer (LT). a, Total Ion Images (TIIs) for m/z = 128 amu from three samples: Green River Shale, Aguas Zarcas (AZ), and Murchison, highlighting three detected peaks (indicated by dashed rectangles). Peaks refer to triplets (m/z,RT1, RT2) with high intensity in their corresponding TII. **b**, To use peaks in machine-learning downstream tasks, we account for small retention time variations. Peaks occurring within a specified maximum distance threshold for RT1 ($RT1_{thrsh}$) and RT2 ($RT2_{thrsh}$), while having the same m/z, are clustered into a single feature. The combined TII demonstrates the consolidated peak features, with the corresponding positions mapped in a features table. **c**, Samples are then classified using logistic regression, and the regression coefficients are ranked by their absolute values. Many top features have close $RT1$ and $RT2$ but different m/z, which may correspond to fragment ions of a parent compound. **d**, First, we sorted the features in descending order based on the absolute value of their regression coefficients. Starting with the feature that had the highest absolute coefficient, we identified all features within a maximum distance of 50 seconds in RT1 and 0.8 in RT2, with varying m/z values, grouped them into a single group, and removed them from the ranked features list. We selected the feature with the highest coefficient in each group as the representative feature. We repeated this process for subsequent groups until no features remained. We ranked each group based on the regression coefficient of its representative feature.

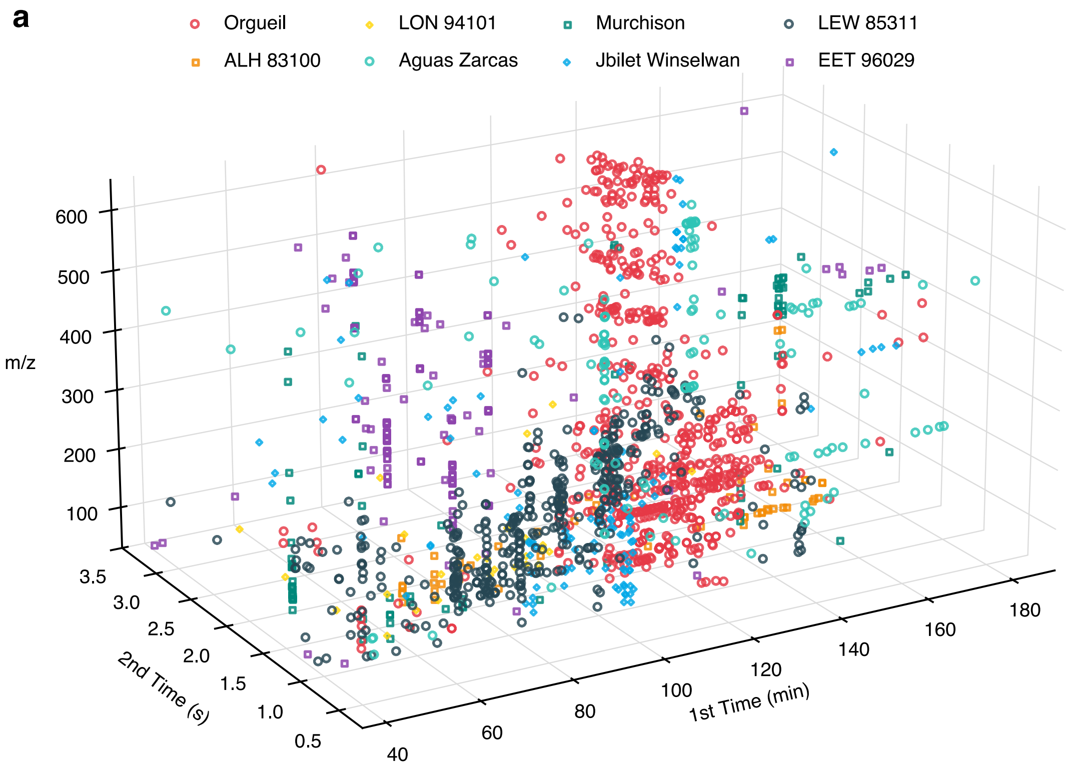

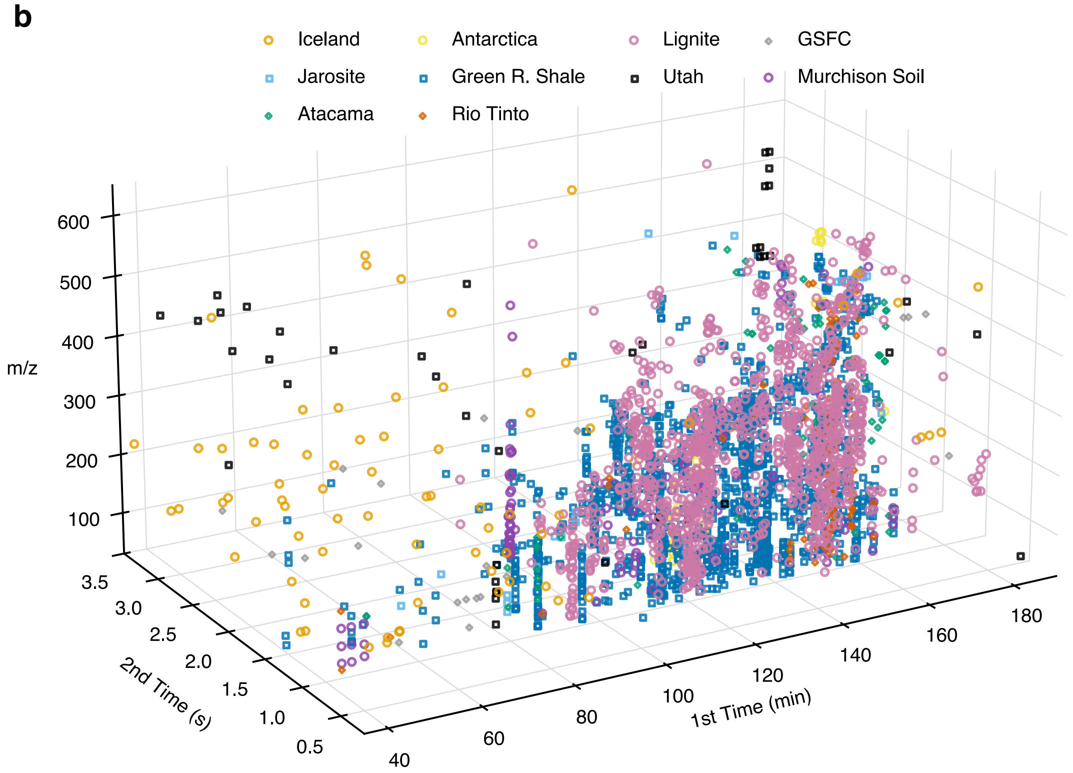

Fig. S5. Visualization of peaks detected by LifeTracer. Distribution of peaks across **(a)** abiotic and **(b)** biotic samples, plotted by their mass-to-charge ratio (m/z), first retention time (RT1), and second retention time (RT2). The abiotic samples contain 9475 peaks, while the biotic samples contain 9070 peaks. Note that the number of peaks does not always indicate the number of distinct compounds; some peaks may represent fragment ions originating from the same parent compound. See Table S3 for more exact number of peaks per sample.

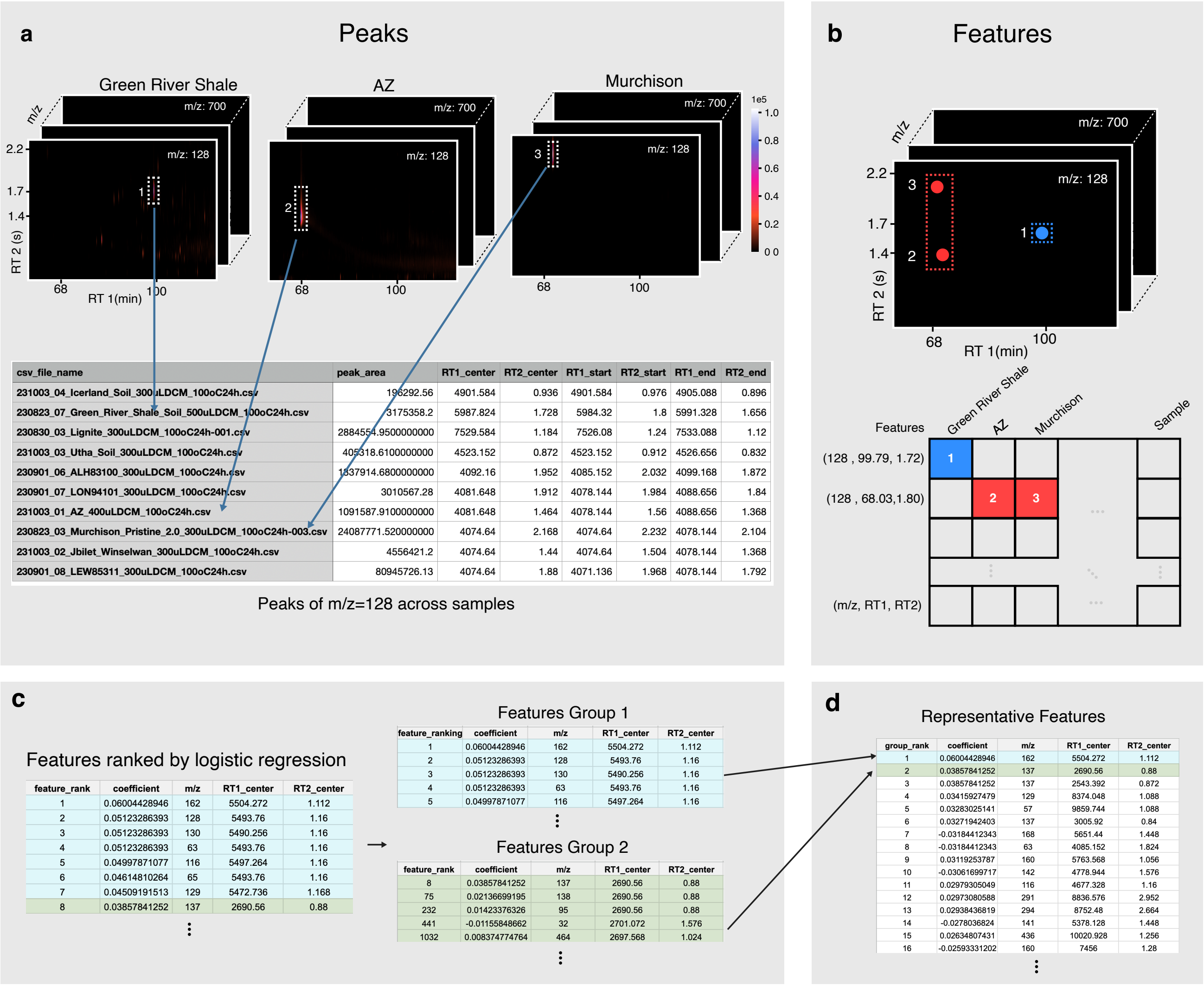

Fig. S6. Comparing peaks, features, feature groups, and representative features. a, Peaks refer to triplets (m/z,RT1,RT2) with high intensity in their corresponding TII. Each peak can represent fragment ion of a parent compound. **b,** In order to use the peaks in down-stream machine-learning tasks, samples containing the same peak should share a feature. However, due to slight variations in RT1 and RT2, we cluster peaks that satisfy the condition mentioned in Figure S4. These clusters form features, where a feature will be 1 if the sample contains that peak and 0 otherwise. **c,** In order to analyze the top features in a systematic manner, we take features with close retention times but varying m/z (which may indicate fragments of a parent compound) are grouped together, forming a feature group based on the procedure in Figure S4. **d,** The feature with the highest regression coefficient is selected as the representative for that group, and we refer to these as representative features.

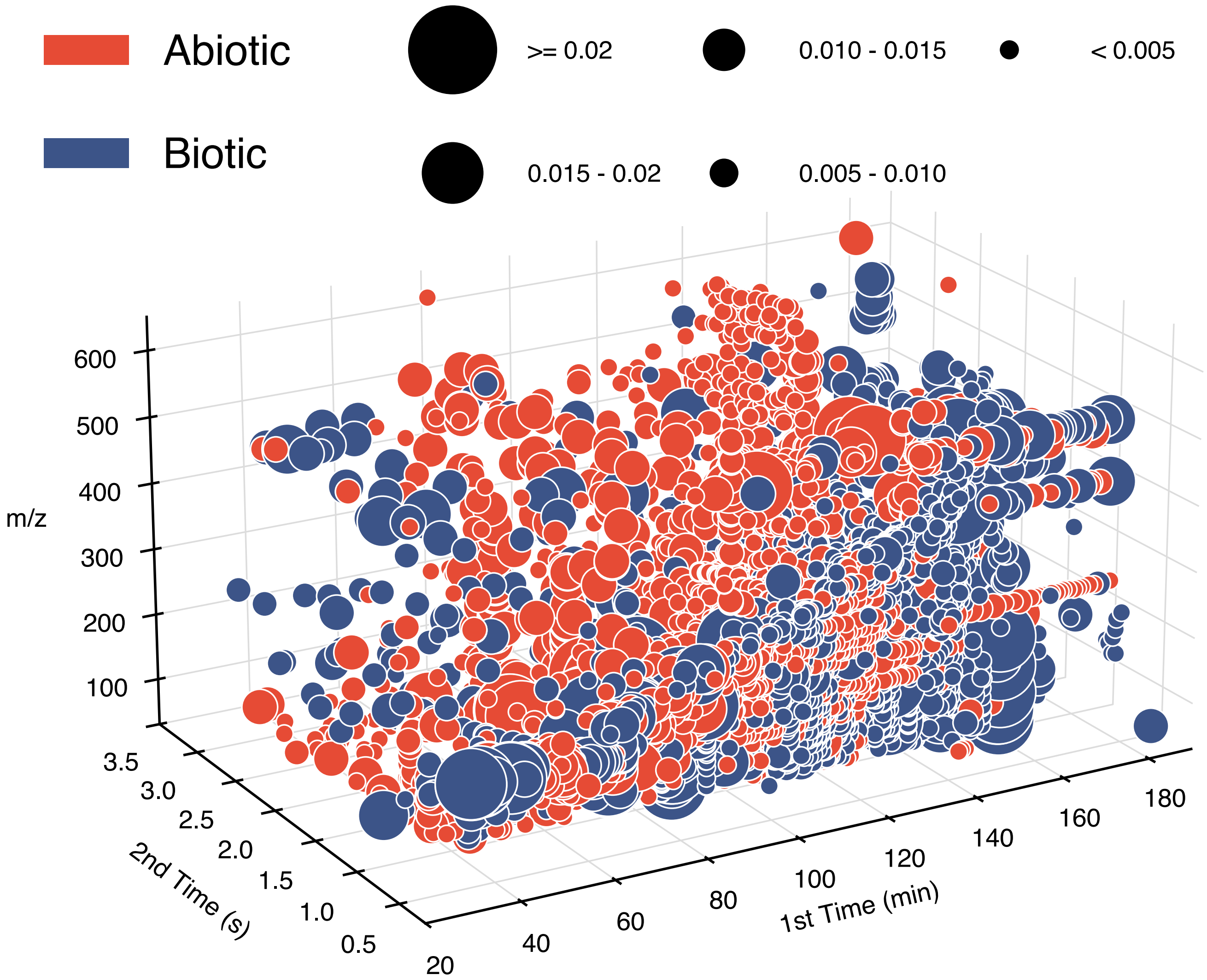

Fig. S7. Visualization of features. Scatter plot showing an inventory of unbiased predictive features in our logistic regression model, LT-Reg. The size of the spheres represents the magnitude of the regression coefficients (importance in classification), and the color indicates the direction of enrichment: red for abiotic and blue for biotic samples.

Daniel Saeedi*, Denise Buckner*, Thomas A. Walton, José C. Aponte, and Amirali Aghazadeh

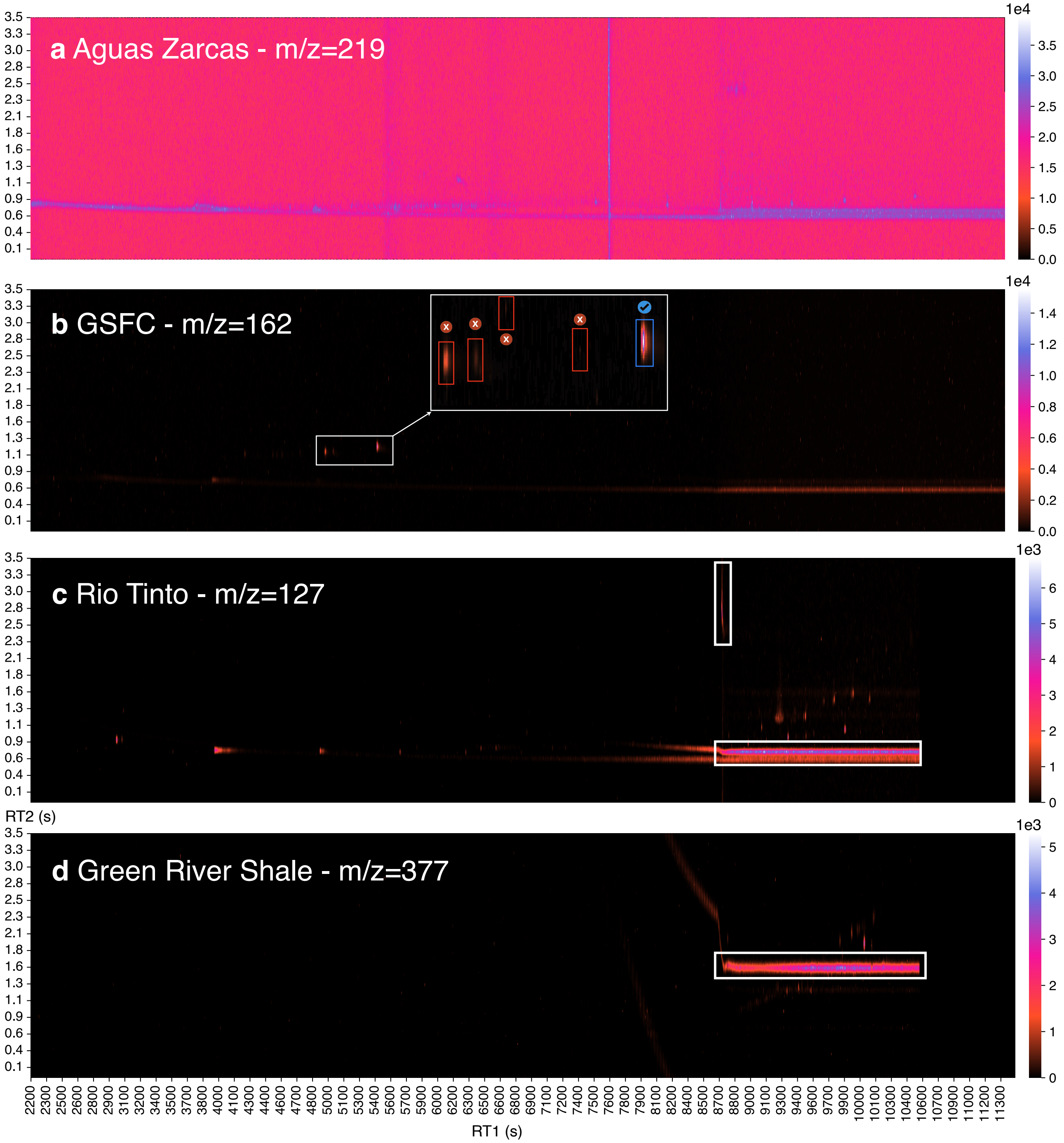

Fig. S8. Examples of noisy TIIs. **a**, TIIs characterized by high-intensity pixels uniformly distributed across the image were identified as noise. The TII for Aguas Zarcas is an example of this noise pattern. **b**, The total intensity of each peak was calculated as the summation of intensities within defined rectangular regions (I_{rect}). Peaks with $I_{\text{rect}} < \lambda_2 \sigma$ were discarded, with $\lambda_2 = 100.0$ providing a balance between retaining relevant data and avoiding the inclusion of noise. **c**,**d**, Vertical and horizontal strip regions on the TIIs were also classified as noise, with examples for Rio Tinto and Green River Shale. *Box colors:* white boxes delineate the regions of the TII being discussed; in **b**, the blue box marks peaks that are retained (not filtered), whereas the red boxes mark peaks that are removed by the filter.

a

Sample	Naphthalene	Biphenyl	Phenanthrene	Anthracene	1-Phenylnaphthalene	Acenaphthene
ALH 83100	1	1	1	0	0	1
Aguas Zarcas	1	0	0	0	0	1
EET 96029	0	0	0	0	0	0
Jbilet Winselwan	1	1	0	0	0	0
Murchison	1	1	1	0	0	1
Orgueil	1	1	1	0	0	0
LEW 85311	1	1	1	1	1	1
LON 94101	1	1	1	0	0	1

b

λ_1	λ_2	Accuracy	# Peaks
1	1	0.92	1697
1	10	0.92	1696
		⋮	
5	100	0.92	494
		⋮	
20	200	0.6	119

c

Sample	$RT1_{center}$	$RT2_{center}$
Orgueil	5195.920	1.848
ALH83100	5202.928	1.664
LON94101	5202.928	1.632
Jbilet Winselwan	5188.912	1.280
LEW 85311	5178.400	1.648

d

Compound	$\Delta RT1$	$\Delta RT2$
Naphthalene	49.056	0.776
Biphenyl	24.528	0.568
Phenanthrene	28.032	0.816
Anthracene	28.032	0.816
1-Phenylnaphthalene	28.032	0.816
Acenaphthene	10.512	0.600

Fig. S9. Automatic calibration of peak detection and clustering parameters using expert-verified reference compounds. a, Expert-verified reference set showing presence (1) or absence (0) of six polycyclic aromatic hydrocarbons across eight meteorite samples. This truth table serves as the ground truth for optimizing peak detection parameters λ_1, λ_2, and clustering thresholds $RT1_{thrsh}$ and $RT2_{thrsh}$. **b,** Grid search results over $\lambda_1 \in \{1, \dots, 20\}$ and $\lambda_2 \in \{1, 10, 20, \dots, 200\}$. For each parameter pair, the peak-detection procedure (described in Methods) was executed and accuracy measured by checking whether each reference compound was successfully recovered within tolerance windows of ± 50 s in RT1 and ± 1 s in RT2. To avoid parameter settings that admit excessive spurious peaks (making manual inspection impractical), we adopted a conservative strategy: among configurations achieving $> 90\%$ accuracy, we selected the largest λ_1 for maximum noise suppression, then chose the median λ_2 at that λ_1 to avoid being either too restrictive (missing real peaks) or too permissive (accepting noise). This procedure yielded optimal parameters $\lambda_1^* = 5$ and $\lambda_2^* = 100$ (blue row), detecting 494 peaks total across all samples and compounds of the reference compounds in part (a) **c,** Example retention time variability for biphenyl across five meteorite samples where it was detected. Each row shows the sample name and the RT1 and RT2 coordinates of the detected biphenyl peak center. Green boxes highlight the minimum (5178.400 s) and maximum (5202.928 s) RT1 values, demonstrating the retention time drift across different samples that must be accommodated by the clustering algorithm. **d,** Retention time dispersions calculated for all six reference compounds to establish data-driven clustering thresholds. Dispersions were computed as $\Delta RT1 = \max(RT1_{center}) - \min(RT1_{center})$ and $\Delta RT2 = \max(RT2_{center}) - \min(RT2_{center})$ across all samples where each compound was detected. For example, biphenyl's RT1 dispersion: $\Delta RT1 = 5202.928 - 5178.400 = 24.528$ s. The maximum dispersions across all compounds (red boxes) represent worst-case retention time variability in the calibration set and were selected as conservative clustering thresholds: $RT1_{thresh} = \max(\Delta RT1) = 49.056 \approx 50$ s (rounded to nearest second) and $RT2_{thresh} = \max(\Delta RT2) = 0.816 \approx 0.8$ s (rounded to 0.1 s). These thresholds ensure the clustering algorithm accommodates instrument drift and sample-to-sample variability while maintaining compound identification accuracy.

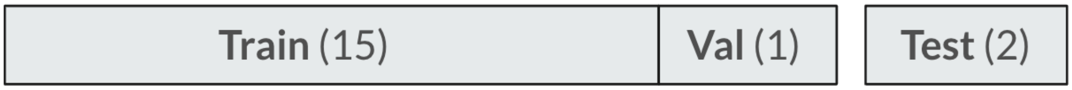

Fig. S10. Schematic representation of the 9-fold cross-validation process used in training and evaluation. For each of the ten different random seeds, the data is split into training, validation, and test sets.

Table S1. Abiotic and biotic samples selected for this study.

**Carbonaceous chondrites.** These "abiotic" samples contain extraterrestrial organic molecules formed through non-biological astrochemical processes. Meteorites provide valuable insights into the chemical inventory of the early solar system.
1) Murchison meteorite. This CM2 chondrite is the "gold standard" for the analysis of extraterrestrial organics; it has a large molecular inventory and low terrestrial contamination.<br>2) Orgueil meteorite. Its chemical composition is almost identical to the Sun's photosphere (1). It is organically and mineralogically distinct from Murchison (2, 3).<br>3) ALH 83100, 4) LON 94101, 5) LEW 85331, and 6) EET 96029 meteorites. CM Antarctic meteorites with organic distributions shaped by varying processing (4).<br>7) Aguas Zarcas (AZ) and 8) Jbilet Winselwan meteorites. CM2 meteorites are similar to Murchison but with different levels of processing and terrestrial contamination.
**Geologically processed samples and soils.** We classify these samples as "biotic" because they harbor of organic compound fossils and relics or current biological species.
9) Lignite. These are types of unrefined minerals and liquids formed from the accumulation and partial decay of organic material over time.<br>10) Green River Shale. The shale in the Green River Formation is an Eocene-aged and organics-rich lithology containing abundant preserved biosignatures from diverse origins.<br>11) Antarctica and 12) Atacama Desert. These samples contain minimal biological activity; their low organic content is key for comparisons against abiotic samples.<br>13) Rio Tinto soil, 14) Jarosite soil. These samples represent acidic, salty, oxidizing, sulfuric, and iron-rich lithologies that can impact organic preservation (5–8).<br>15) Murchison Soil, 16) Utah, 17) GSFC, and 18) Iceland soils. Murchison soil serves as a contamination check for the Murchison meteorite, and Utah/GSFC/Iceland represents organics-rich soils hosting active modern microbial communities.

Table S2. Comparison of model accuracies for five classifiers. Values are mean ± standard deviation across seeds.

Model	Validation Accuracy (%)	Test Accuracy (%)
Random Classifier	55	55
Random Forest	81 ± 1	78 ± 3
Support Vector Machine (SVM)	80 ± 0	73 ± 5
Bernoulli Naive Bayes	76 ± 1	75 ± 7
Logistic Regression with L1 regularization	93 ± 1	84 ± 5
Logistic Regression with L2 regularization	91 ± 0	87 ± 5

Table S3. **Biotic and abiotic samples with their raw file size and number of detected peaks.** **The table includes 18 samples, comprising terrestrial soils and meteorites, classified as either biotic or abiotic. The size (in gigabytes, G) represents the data volume of each sample, while the number of peaks corresponds to the peaks detected by LifeTracer for each sample.**

#	Sample	Category	Size	# peaks
1	Atacama Soil	Biotic	14 G	214
2	Rio Tinto Soil	Biotic	13 G	363
3	Murchison Soil	Biotic	13 G	402
4	Antarctica Soil	Biotic	12 G	209
5	Jarosite Soil	Biotic	11 G	66
6	Green River Shale Soil	Biotic	18 G	3543
7	Lignite	Biotic	25 G	3645
8	GSFC Soil	Biotic	18 G	137
9	Utah Soil	Biotic	15 G	228
10	Iceland Soil	Biotic	13 G	263
11	Murchison	Abiotic	13 G	288
12	ALH 83100	Abiotic	20 G	715
13	LON 94101	Abiotic	18 G	346
14	LEW 85311	Abiotic	24 G	1923
15	Aguas Zarcas (AZ)	Abiotic	19 G	593
16	Jbilet Winselwan	Abiotic	15 G	722
17	EET 96029	Abiotic	22 G	336
18	Orgueil	Abiotic	29 G	4552

Table S4. Performance comparison of machine learning classifiers using nested stratified K-fold cross-validation. Values represent mean AUC ± standard deviation across 10 random seeds with 6-fold outer and 5-fold inner cross-validation. AUC values closer to 1.0 indicate better performance.

Model	AUC of Validation	AUC of Test
Random Forest	1.00 ± 0.00	0.79 ± 0.05
Support Vector Machine (SVM)	0.90 ± 0.04	0.94 ± 0.06
Bernoulli Naive Bayes	0.83 ± 0.03	0.89 ± 0.10
Logistic Regression with L1 regularization	0.99 ± 0.01	0.92 ± 0.06
Logistic Regression with L2 regularization	0.98 ± 0.01	0.93 ± 0.07

Table S5. A complete list of feature groups with their corresponding representative feature. The coefficient column reflects the logistic regression's assigned weight for each representative feature, while the samples column lists the samples in which these representative features are present. The RT1 and RT2 columns display the range of retention times covered by the representative feature. The identities of these molecules were manually confirmed by comparing their mass fragmentation patterns and matching their retention times to standards, as listed in the Identified Compound column. Note that some compounds identified by *LifeTracer* might be noise, which is indicated by the expert in the Identified Compound column. The white color represents compounds with exact matches in both structure and identification, while darker grey indicates cases where multiple isomers are possible, allowing for identification by compound class rather than precise structure. Light blue indicates unknown compounds with a possible molecular class assignment, while dark blue indicates completely unknown compounds. Yellow color represents sulfur background. Red color represents no peak found or noise. The feature groups with the same magnitude (absolute value) of regression coefficients, their ranking are the same.

Rank	Coef	m/z	RT1	RT2	Samples	Identified Compound
1	0.0600	162	[5483.248, 5525.296]	[1.288, 0.936]	Iceland, Jarosite, Atacama, Lignite, Utah, GSFC, Murchison Soil, ALH 83100	A polysubstituted C6-alkylbenzene with the formula C12H18. Peak not found in Lignite (noise).
2	0.0386	137	[2690.56, 2690.56]	[0.92, 0.84]	Iceland, Atacama, Rio Tinto, Utah, Murchison Soil	Possibly 4a-methyldecahy dronaphthalene or a structural isomer. Found in all samples.
2	0.0386	137	[2539.888, 2546.896]	[0.912, 0.832]	Iceland, Atacama, Rio Tinto, Utah, Murchison Soil	Possibly 4a-methyldecahy dronaphthalene or a structural isomer. Found in all samples.
3	0.0342	129	[8360.032, 8388.064]	[1.128, 1.048]	Jarosite, Antarctica, GSFC, Murchison Soil, ALH 83100, LON 94101, LEW 85311	Possibly diisooctyl adipate or stigmas-5-ene-3β-ol. Peak too low in abundance in Murchison Soil for peak detection.
4	0.0328	57	[9856.24, 9863.248]	[1.136, 1.048]	Atacama, Green R. Shale, Rio Tinto, Lignite, GSFC, Murchison Soil	n-x, found in all samples.
5	0.0327	137	[3005.92, 3009.424]	[0.888, 0.8]	Iceland, Atacama, Utah, Murchison Soil	Possibly 4a-methyldecahydronaphthalene or a structural isomer. Found in all samples.
6	-0.0318	168	[5630.416, 5675.968]	[1.688, 1.2]	Orgueil, ALH 83100, LON 94101, Aguas Zarcas, Murchison, Jbilet Winselwan, LEW 85311	Possible methyl biphenyl or another isomer with the formula C13H12. Only detected in Jbilet Winselwan and LEW 85311, different compound in ALH 83100, not found in remaining samples (noise).
6	-0.0318	63 *	[4081.648, 4092.16]	[2.192, 1.456]	Orgueil, ALH 83100, LON 94101, Aguas Zarcas, Murchison, Jbilet Winselwan, LEW 85311	Naphthalene, found in all samples
7	0.0312	160	[5742.544, 5788.096]	[1.096, 1.008]	Jarosite, Lignite, GSFC	Unknown compound found in GSFC and Lignite. Different unknown compound in Jarosite.
8	-0.0306	142	[4754.416, 4803.472]	[1.856, 1.296]	Green R. Shale, Orgueil, ALH 83100, LON 94101, Aguas Zarcas, Murchison, Jbilet Winselwan, LEW 85311	2-methylnaphthalene, detected in all samples.
9	0.0298	116 †	[4673.824, 4677.328]	[1.296, 1.024]	Jarosite, Utah	C5-alkylbenzene with the formula C11H16. Found in all samples.
10	0.0297	291	[8819.056, 8854.096]	[3.24, 2.656]	Jarosite, Atacama, Antarctica, Green R. Shale, Rio Tinto, Murchison Soil, Murchison, EET 96029	Possible phosphine oxide, diphenyl(phenylmethyl)- detected in three samples. Not found in remaining samples (noise).
11	0.0294	294	[8727.952, 8773.504]	[2.696, 2.624]	Jarosite, Rio Tinto, Murchison Soil	Not found in any samples (noise).
12	-0.0278	141	[5357.104, 5399.152]	[1.688, 1.208]	Green R. Shale, Lignite, ALH 83100, LON 94101, Aguas Zarcas, Murchison, Jbilet Winselwan, LEW 85311	C2-alkylnaphthalene with the formula C12H12. Found in all samples except in Aguas Zarcas (noise).
13	0.0263	436	[10003.408, 10034.944]	[1.256, 1.248]	Jarosite, Atacama	Not found in any samples (noise).

*In this feature group, the fragment ions of this compound with m/z 63, 101, 102, 127, and 129 share the same regression coefficient. Any of these can serve as the representative feature for the group.
†In this feature group, the fragment ions of this compound with m/z 63, 65, and 116 share the same regression coefficient. Any of these can serve as the representative feature for the group.

Rank	Coef	m/z	RT1	RT2	Samples	Identified Compound
14	-0.0259	160	[7431.472, 7477.024]	[1.52, 1.048]	Green R. Shale, Orgueil, ALH 83100, LON 94101, Aguas Zarcas, Murchison, LEW 85311	Sulfur background in 4 samples, unknown compound in one sample, not found in LON 94101 and Aguas Zarcas (noise).
15	-0.0251	115	[4842.016, 4849.024]	[1.872, 1.312]	Orgueil, ALH 83100, LON 94101, Murchison, Jbilet Winselwan, LEW 85311	Substituted cycloalkane with the formula C11H10. Found in all samples.
16	-0.0244	464	[6565.984, 6594.016]	[1.216, 1.008]	Orgueil, Aguas Zarcas, Jbilet Winselwan, EET 96029	Not found in any samples (noise).
16	0.0244	162	[4999.696, 5003.2]	[1.2, 1.136]	Atacama, GSFC, Murchison Soil	C6-alkylbenzene with the formula C12H18. Found in all samples.
17	-0.0243	168	[6061.408, 6110.464]	[1.752, 1.24]	Lignite, ALH 83100, LON 94101, Aguas Zarcas, Murchison, LEW 85311	C3-alkylnaphthalene with the formula C13H12, likely isopropenylpahthalene. Found in two samples, a different compound in Lignite, not found in LON 94101, ALH 83100, or EET 96029 (noise).
17	-0.0243	152	[5171.392, 5202.928]	[2.152, 1.64]	Orgueil, ALH 83100, LON 94101, LEW 85311, EET 96029	C2-naphthalene with the formula C12H10, likely 2-ethenylnaphthalene. Found in two samples, not found in LON 94101, ALH 83100, and EET 96029 samples (noise).
17	0.0243	469	[9463.792, 9502.336]	[1.28, 1.248]	Jarosite, Rio Tinto	Not found in any samples (noise).
17	0.0243	233	[6303.184, 6306.688]	[1.192, 1.176]	Jarosite, Rio Tinto	Unknown compound in Rio Tinto sample, not found in other sample (noise).
18	0.0224	365	[9372.688, 9390.208]	[1.176, 1.16]	Rio Tinto, GSFC, Murchison Soil	Polycyclic terpenoid with an unidentified structure. Found in Rio Tinto samples, not found in Murchison soil and GSFC (noise).
19	-0.0221	464	[8552.752, 8601.808]	[1.912, 1.024]	ALH 83100, Aguas Zarcas, EET 96029	Not found in any samples (noise).
20	0.0218	182	[7561.12, 7564.624]	[1.36, 1.176]	Antarctica, Utah	Unknown compound in Antarctica sample, not found in other sample (noise).
21	-0.0208	464	[8493.184, 8528.224]	[2.176, 1.128]	Lignite, ALH 83100, Aguas Zarcas, EET 96029	Not found in any samples (noise).
22	0.0199	36	[2200.0, 2207.008]	[2.088, 1.08]	Jarosite, Atacama, Antarctica, Lignite, Aguas Zarcas, EET 96029	Not found in any samples (noise).
23	-0.0198	464	[8079.712, 8125.264]	[1.672, 1.016]	Orgueil, Aguas Zarcas, EET 96029	Not found in any samples (noise).
23	-0.0198	464	[8058.688, 8100.736]	[3.232, 2.56]	Orgueil, Aguas Zarcas, EET 96029	Not found in any samples (noise).
24	0.0197	464	[2462.8, 2511.856]	[3.24, 2.736]	Iceland, Utah	Not found in any samples (noise).
24	0.0197	464	[5101.312, 5125.84]	[1.392, 1.36]	Iceland, Utah	Not found in any samples (noise).
24	0.0197	374	[11373.472, 11419.024]	[1.048, 1.04]	Iceland, Utah	Not found in any samples (noise).
24	0.0197	464	[2273.584, 2287.6]	[1.888, 1.408]	Iceland, Utah	Not found in any samples (noise).
24	0.0197	464	[2830.72, 2851.744]	[1.904, 1.36]	Iceland, Utah	Not found in any samples (noise).
24	0.0197	464	[4519.648, 4554.688]	[1.832, 1.312]	Iceland, Utah	Not found in any samples (noise).
25	0.0193	111	[4288.384, 4291.888]	[1.064, 1.04]	Atacama, GSFC	Unknown compound found in both samples.

Rank	Coef	m/z	RT1	RT2	Samples	Identified Compound
25	0.0193	83	[4432.048, 4442.56]	[1.152, 1.112]	Atacama, GSFC	Unknown compound found in both samples.
25	0.0193	144	[4905.088, 4908.592]	[1.144, 1.088]	Atacama, GSFC	Tetramethylindane or tetrahydrotrimethylnaphthalene, found in both samples.
25	-0.0193	370	[8945.2, 8976.736]	[1.552, 1.416]	Murchison, EET 96029	Siloxane in found in Murchison, not found in EET 96029 (noise).
26	-0.0192	216	[6187.552, 6233.104]	[1.08, 1.008]	Orgueil, ALH 83100, LON 94101, Murchison	Not found in any samples (noise).
27	-0.0190	106	[5048.752, 5059.264]	[1.136, 1.04]	ALH 83100, LON 94101, Murchison, LEW 85311	Same Unknown compound in Murchison and LEW 85311 samples, two different unknown compounds in other two samples.
27	-0.0190	92	[3314.272, 3352.816]	[1.136, 1.072]	ALH 83100, LON 94101, Murchison, LEW 85311	Possible C4-alkylbenzene (C10H14) or benzenepropanal (C9H14O), but different unknown compound in LEW 85311 sample.
27	-0.0190	92 ‡	[3934.48, 3952.0]	[1.104, 1.048]	ALH 83100, LON 94101, Murchison, LEW 85311	C5-alkylbenzene with the formula C11H16. Too low in abundance for detection in Murchison sample.
27	-0.0190	92	[4379.488, 4390.0]	[1.12, 1.072]	ALH 83100, LON 94101, Murchison, LEW 85311	C5-alkylbenzene with the formula C11H16. Found in three samples, different unknown compound in ALH 83100 sample.
28	0.0184	440	[9092.368, 9092.368]	[3.192, 3.192]	Jarosite	-
28	0.0184	412	[10301.248, 10301.248]	[3.152, 3.152]	Jarosite	-
28	0.0184	456	[10280.224, 10280.224]	[1.576, 1.576]	Jarosite	-
28	0.0184	456	[10087.504, 10087.504]	[1.576, 1.576]	Jarosite	-
28	0.0184	454	[9719.584, 9719.584]	[1.28, 1.28]	Jarosite	-
28	0.0184	130	[5322.064, 5322.064]	[1.136, 1.136]	Jarosite	-
28	0.0184	123	[3447.424, 3447.424]	[1.056, 1.056]	Jarosite	-
28	0.0184	456	[10479.952, 10522.0]	[1.584, 1.56]	Jarosite	-
28	0.0184	456	[10346.8, 10346.8]	[1.584, 1.584]	Jarosite	-
28	-0.0184	464	[8615.824, 8647.36]	[2.304, 1.08]	Lignite, Orgueil, Aguas Zarcas, EET 96029	Not found in any samples (noise).
29	0.0182	464	[7151.152, 7193.2]	[2.704, 2.112]	Iceland, Utah, Orgueil	Not found in any samples (noise).
30	-0.0181	160	[7371.904, 7420.96]	[1.64, 1.064]	Lignite, Orgueil, ALH 83100, LON 94101, Aguas Zarcas, LEW 85311	Sulfur background in 4 samples, not found in Lignite and LON 94101 samples (noise).
31	-0.0180	118	[3146.08, 3184.624]	[1.328, 1.112]	ALH 83100, LON 94101, Aguas Zarcas, LEW 85311	Indane
31	-0.0180	124	[3528.016, 3542.032]	[2.6, 1.912]	ALH 83100, LON 94101, Aguas Zarcas, LEW 85311	1,2,4-trithiolane
32	-0.0175	139	[5244.976, 5294.032]	[2.192, 1.2]	Jbilet Winselwan, LEW 85311, EET 96029	Dimethylnaphthalene. Not found in EET 96029 (noise).
33	0.0169	245	[7224.736, 7231.744]	[1.168, 1.008]	Antarctica, Lignite, Murchison Soil	Polycyclic terpenoid with an unidentified structure. Found in two samples, not found in one (noise).

‡In this feature group, the fragment ions of this compound with m/z 92 and 106 share the same regression coefficient. Any of these can serve as the representative feature for the group.

Rank	Coef	m/z	RT1	RT2	Samples	Identified Compound
34	0.0163	172	[9751.12, 9793.168]	[1.488, 1.264]	Antarctica, Rio Tinto	Possible Stigmastan-6,22-dien,3,5-dedihydro in Rio Tinto sample, not found in the other sample (noise).
34	0.0163	42	[9071.344, 9074.848]	[1.104, 1.08]	Atacama, Green R. Shale, Rio Tinto, Lignite	Three different unknown compounds in three samples, not found in Atacama sample (noise).
34	0.0163	137	[2760.64, 2760.64]	[0.888, 0.808]	Iceland, Atacama	Possibly 4a-methyldecahydronaphthalene or a structural isomer. Found in all samples.
35	-0.0159	108	[3853.888, 3857.392]	[1.344, 1.064]	Orgueil, Aguas Zarcas, Murchison	Unknown compound, likely with the formula $C_{10}H_{16}O$. Found in all samples.
36	0.0156	217	[7133.632, 7140.64]	[1.096, 1.088]	Antarctica, Murchison Soil	Unknown compound in Antarctica sample, not found in other sample (noise).
37	0.0154	132	[6874.336, 6919.888]	[1.152, 1.072]	Antarctica, Lignite, Murchison Soil, Orgueil	Unknown compound in Antarctica, Murchison Soil, and Orgueil samples, not found in Lignite sample (noise).
38	-0.0151	91	[4022.08, 4025.584]	[1.064, 1.008]	LON 94101, Murchison, LEW 85311	C6-alkylbenzene with the formula $C_{12}H_{18}$. Found in all samples.
39	0.0150	168	[7841.44, 7844.944]	[1.6, 1.2]	Utah, Murchison Soil, Orgueil	Unknown compound with the formula $C_{18}H_{22}$ in Orgueil sample only, not found in other samples (noise).
40	0.0149	212	[6488.896, 6523.936]	[1.336, 1.032]	Iceland, Lignite, Utah, Orgueil, Jbilet Winselwan	Possible diisopropylnaphthalene found in three samples, not found in Utah and Lignite.
40	-0.0149	170	[5875.696, 5914.24]	[1.6, 1.192]	Green R. Shale, Lignite, Orgueil, ALH 83100, LON 94101, Jbilet Winselwan, LEW 85311	Trimethylnaphthalene. Found in all samples, except ALH 83100,Green River Shale, and Lignite samples (noise).
40	-0.0149	141	[5441.2, 5458.72]	[1.704, 1.24]	Green R. Shale, Lignite, Orgueil, ALH 83100, LON 94101, Jbilet Winselwan, LEW 85311	C2-alkylnaphthalene with the formula $C_{12}H_{12}$. Found in all samples.
40	-0.0149	170	[5798.608, 5840.656]	[1.576, 1.136]	Green R. Shale, Lignite, Orgueil, ALH 83100, LON 94101, Jbilet Winselwan, LEW 85311	Trimethylnaphthalene. Found in all samples except LON 94101 and Orgueil (noise).
40	-0.0149	184	[6131.488, 6177.04]	[1.464, 1.136]	Lignite, Orgueil, ALH 83100, LON 94101, Jbilet Winselwan	Different compounds in Lignite and Orgueil samples, not found in other samples (noise).
41	-0.0146	170	[5942.272, 5952.784]	[1.616, 1.216]	Lignite, ALH 83100, LON 94101, Jbilet Winselwan, LEW 85311	Trimethylnaphthalene. Found in all samples, except Jbilet Winselwan and ALH 83100 (noise).
42	0.0143	65	[3072.496, 3083.008]	[1.184, 1.16]	Jarosite, Murchison Soil, Orgueil, Murchison	C4-alkylbenzene with the formula $C_{10}H_{14}$. Found in all samples.
43	-0.0141	91 §	[2385.712, 2420.752]	[1.328, 1.096]	LON 94101, Aguas Zarcas, LEW 85311	C3-alkylbenzene with the formula C_9H_{12}. Found in three samples.
43	-0.0141	453	[10539.52, 10581.568]	[1.24, 1.072]	LON 94101, Aguas Zarcas, LEW 85311	Not found in any samples (noise).
43	0.0141	145	[7049.536, 7077.568]	[1.064, 1.024]	Antarctica, Murchison Soil, Orgueil	Different unknown compounds in Antarctica and Murchison Soil sample, not found in Orgueil sample (noise).
43	0.0141	170	[6758.704, 6790.24]	[1.144, 1.008]	Antarctica, Murchison Soil, Orgueil	Different unknown compounds in Antarctica and Murchison Soil sample, not found in Orgueil sample (noise).
43	0.0141	288	[7732.816, 7781.872]	[1.336, 1.008]	Antarctica, Murchison Soil, Orgueil	Not found in any samples (noise).

§ In this feature group, the fragment ions of this compound with m/z 91 and 120 share the same regression coefficient. Any of these can serve as the representative feature for the group.

Rank	Coef	m/z	RT1	RT2	Samples	Identified Compound
44	-0.0139	146	[2967.376, 3009.424]	[1.992, 1.32]	Orgueil, Murchison, Jbilet Winselwan	Dichlorobenzene. Found in all samples, except in Jbilet Winselwan (noise).
45	0.0136	212	[6348.736, 6394.288]	[1.264, 1.016]	Iceland, Utah, Orgueil, Jbilet Winselwan	Not found in any samples (noise).
46	0.0131	369	[10220.656, 10269.712]	[2.712, 2.128]	Antarctica, Green R. Shale, GSFC, ALH 83100, LON 94101	Not found in any samples (noise).
46	-0.0131	230	[6667.6, 6706.144]	[1.12, 1.016]	Orgueil, ALH 83100, Murchison	Not found in any samples (noise).
47	-0.0130	464	[6793.744, 6842.8]	[2.432, 1.952]	Orgueil, EET 96029	Not found in any samples (noise).
47	-0.0130	464	[7420.96, 7463.008]	[3.352, 2.464]	Orgueil, EET 96029	Not found in any samples (noise).
47	0.0130	160	[6923.392, 6972.448]	[1.216, 1.08]	Antarctica, Green R. Shale, Lignite	Not found in any samples (noise).
47	0.0130	467	[11264.848, 11313.904]	[1.104, 1.048]	Iceland, Utah, Aguas Zarcas	Not found in any samples (noise).
47	0.0130	467	[11068.624, 11110.672]	[1.08, 1.064]	Iceland, Utah, Aguas Zarcas	Not found in any samples (noise).
47	0.0130	467	[10893.424, 10935.472]	[1.08, 1.048]	Iceland, Utah, Aguas Zarcas	Not found in any samples (noise).
47	0.0130	467	[10763.776, 10812.832]	[1.08, 1.056]	Iceland, Utah, Aguas Zarcas	Not found in any samples (noise).
47	0.0130	467	[10627.12, 10676.176]	[1.08, 1.064]	Iceland, Utah, Aguas Zarcas	Not found in any samples (noise).
47	-0.0130	200	[7508.56, 7554.112]	[2.624, 2.136]	Lignite, Orgueil, ALH 83100, Murchison, LEW 85311	Fluoranthene. Found in all samples, except Lignite (noise)
48	-0.0129	464	[6530.944, 6572.992]	[2.392, 1.616]	Orgueil, Aguas Zarcas, Jbilet Winselwan	Not found in any samples (noise).
48	-0.0129	132	[5560.336, 5602.384]	[1.648, 1.032]	LON 94101, Aguas Zarcas	Not found in any samples (noise).
49	0.0128	150	[8247.904, 8251.408]	[2.464, 2.44]	Lignite, GSFC	Not found in any samples (noise).
50	-0.0127	174	[5448.208, 5458.72]	[2.6, 2.224]	Orgueil, ALH 83100, LON 94101, LEW 85311	Unknown compound found in Orgueil, ALH 83100, and LEW 85311 samples, all very low in abundance. Detected peak for LON 94101 is noise.
50	0.0127	182	[7631.2, 7662.736]	[1.344, 1.152]	Lignite, Utah	Not found in any samples (noise).
50	0.0127	263	[8240.896, 8244.4]	[1.48, 1.152]	Lignite, Utah	Not found in any samples (noise).
51	0.0123	382	[9933.328, 9943.84]	[1.56, 1.544]	Rio Tinto, Lignite, Murchison Soil	Unknown compound in Lignite sample, not found in the other samples (noise).
52	0.0122	372	[9253.552, 9292.096]	[1.704, 1.176]	Green R. Shale, Rio Tinto, Murchison Soil	Possible cholestane in Green R. Shale sample, not found in the other two samples (noise).
52	0.0122	382	[9116.896, 9148.432]	[1.4, 1.016]	Green R. Shale, Rio Tinto, Murchison Soil	Not found in any samples (noise).
52	0.0122	372	[9190.48, 9236.032]	[1.704, 1.096]	Green R. Shale, Rio Tinto, Murchison Soil	Possible cholestane in Green R. Shale sample, not found in the other two samples (noise).
53	-0.0121	132	[3478.96, 3528.016]	[1.16, 1.008]	LON 94101, Jbilet Winselwan, LEW 85311	Unidentified cyclic compound in two samples, not found in one sample (noise).
53	-0.0121	171	[5998.336, 6036.88]	[1.472, 1.056]	LON 94101, Jbilet Winselwan, LEW 85311	Trimethylnaphthalene in LON 94101 sample, not found in the other two samples (noise).
53	-0.0121	213	[7687.264, 7722.304]	[1.632, 1.008]	Orgueil, ALH 83100, Aguas Zarcas	Not found in any samples (noise).

Rank	Coef	m/z	RT1	RT2	Samples	Identified Compound
54	0.0118	184	[7336.864, 7340.368]	[1.264, 1.176]	Antarctica, Lignite	Unknown compound in Antarctica sample, not found in the other sample (noise)
55	-0.0116	168	[8875.12, 8889.136]	[1.832, 1.072]	ALH 83100, Murchison	Not found in any samples (noise).
55	-0.0116	113	[5171.392, 5171.392]	[3.184, 3.184]	EET 96029	-
55	-0.0116	123	[5304.544, 5304.544]	[3.072, 3.072]	EET 96029	-
55	-0.0116	530	[4877.056, 4887.568]	[3.104, 2.408]	EET 96029	-
55	-0.0116	529	[5076.784, 5083.792]	[3.264, 2.704]	EET 96029	-
55	-0.0116	32	[2525.872, 2560.912]	[2.528, 1.32]	EET 96029	-
55	-0.0116	32	[2595.952, 2627.488]	[2.232, 2.168]	EET 96029	-
55	-0.0116	32	[5227.456, 5227.456]	[3.072, 3.072]	EET 96029	-
55	-0.0116	614	[3279.232, 3279.232]	[2.136, 2.136]	EET 96029	-
55	-0.0116	647	[10031.44, 10031.44]	[2.816, 2.816]	EET 96029	-
55	-0.0116	32	[2588.944, 2588.944]	[3.448, 3.448]	EET 96029	-
55	-0.0116	231	[2378.704, 2378.704]	[2.112, 2.112]	EET 96029	-
56	-0.0115	184	[6611.536, 6660.592]	[2.336, 2.008]	Green R. Shale, Orgueil, ALH 83100, LON 94101, LEW 85311	Different unknown compounds in Green River Shale and LEW 85311 samples, not found in the other samples (noise).
57	0.0114	171	[11019.568, 11019.568]	[1.112, 1.112]	GSFC	-
57	0.0114	199	[6443.344, 6443.344]	[1.168, 1.168]	GSFC	-
57	0.0114	150	[4824.496, 4824.496]	[2.688, 2.688]	GSFC	-
57	0.0114	150	[8598.304, 8598.304]	[2.632, 2.632]	GSFC	-
57	0.0114	112	[4985.68, 4985.68]	[3.4, 3.4]	GSFC	-
57	0.0114	379	[11142.208, 11142.208]	[1.512, 1.512]	GSFC	-
57	0.0114	112	[4158.736, 4204.288]	[1.032, 1.016]	GSFC	-
57	0.0114	112	[4015.072, 4015.072]	[2.152, 2.152]	GSFC	-
57	0.0114	32	[3710.224, 3710.224]	[1.32, 1.32]	GSFC	-
57	0.0114	150	[8324.992, 8324.992]	[2.064, 2.064]	GSFC	-
57	0.0114	150	[7746.832, 7746.832]	[2.896, 2.896]	GSFC	-
57	0.0114	150	[7010.992, 7010.992]	[3.408, 3.408]	GSFC	-
57	0.0114	150	[6397.792, 6397.792]	[2.368, 2.368]	GSFC	-
57	0.0114	150	[2778.16, 2778.16]	[2.768, 2.768]	GSFC	-
57	-0.0114	201	[7687.264, 7701.28]	[2.728, 2.352]	Lignite, Orgueil, ALH 83100, LON 94101, LEW 85311	Pyrene. Found in all samples, except LON 94101 and Lignite (noise).

Rank	Coef	m/z	RT1	RT2	Samples	Identified Compound
57	-0.0114	453	[11222.8, 11229.808]	[1.08, 1.072]	Aguas Zarcas, Jbilet Winselwan	Not found in any samples (noise).
57	0.0114	370	[10952.992, 10995.04]	[3.296, 3.288]	Utah	-
57	0.0114	371	[10812.832, 10847.872]	[3.312, 3.296]	Utah	-
57	0.0114	371	[10882.912, 10917.952]	[3.304, 3.288]	Utah	-
57	0.0114	464	[2606.464, 2652.016]	[1.376, 1.112]	Utah	-
57	0.0114	464	[2298.112, 2298.112]	[3.072, 3.072]	Utah	-
57	0.0114	464	[2998.912, 2998.912]	[2.936, 2.936]	Utah	-
57	0.0114	464	[3188.128, 3188.128]	[3.24, 3.24]	Utah	-
57	0.0114	464	[3657.664, 3675.184]	[3.344, 3.144]	Utah	-
57	0.0114	464	[3321.28, 3321.28]	[2.952, 2.952]	Utah	-
57	0.0114	464	[5949.28, 5949.28]	[2.648, 2.648]	Utah	-
57	0.0114	371	[11023.072, 11058.112]	[3.312, 3.288]	Utah	-
57	0.0114	220	[6138.496, 6138.496]	[2.848, 2.848]	Utah	-
57	0.0114	277	[8682.4, 8727.952]	[1.712, 1.128]	Utah	-
57	0.0114	464	[2722.096, 2722.096]	[2.008, 2.008]	Utah	-
58	-0.0113	105	[3615.616, 3619.12]	[1.024, 1.016]	ALH 83100, LON 94101, LEW 85311	C5-alkylbenzene with the formula C11H16. Found in all samples, except ALH 83100 (noise).
58	-0.0113	173	[5686.48, 5725.024]	[1.072, 1.016]	ALH 83100, LON 94101, LEW 85311	Not found in any samples (noise).
58	-0.0113	145	[5150.368, 5185.408]	[1.072, 1.04]	ALH 83100, LON 94101, LEW 85311	Not found in any samples (noise).
59	0.0109	382	[9656.512, 9656.512]	[1.456, 1.448]	Rio Tinto, Murchison Soil	Not found in any samples (noise).
60	0.0107	454	[10182.112, 10231.168]	[1.248, 1.24]	Jarosite, Murchison	Possible polycyclic terpenoid with an unidentified structure. Found in all samples.
61	-0.0105	218	[8020.144, 8069.2]	[2.376, 1.632]	Lignite, ALH 83100, Aguas Zarcas, LEW 85311	Possible naphthalene,2-methyl-1-phenyl, or another C6-alkylnaphthalene with the formula C17H14. Found in all samples, except Aguas Zarcas (noise)
61	0.0105	246	[6842.8, 6842.8]	[1.352, 1.352]	Antarctica	-
61	0.0105	369	[10434.4, 10434.4]	[2.128, 2.128]	Antarctica	-
62	0.0104	246	[7879.984, 7911.52]	[1.664, 1.392]	Antarctica, Green R. Shale, LEW 85311	Different unknown compounds in Antarctica and Green River Shale samples, not found in LEW 85311 sample (noise).
63	0.0101	214	[7312.336, 7354.384]	[3.232, 3.128]	Utah, LEW 85311	Not found in any samples (noise).
64	-0.0100	128	[4141.216, 4176.256]	[1.96, 1.912]	ALH 83100, LON 94101	Unidentified polycyclic compound, found in all sampes.
65	0.0099	464	[7224.736, 7273.792]	[2.192, 2.184]	Utah, Orgueil	Not found in any samples (noise).

Rank	Coef	m/z	RT1	RT2	Samples	Identified Compound
66	-0.0095	464	[6720.16, 6737.68]	[3.04, 1.984]	Orgueil, Aguas Zarcas, LEW 85311	Not found in any samples (noise).
67	0.0094	220	[8139.28, 8188.336]	[2.136, 1.224]	Iceland, Green R. Shale, Lignite, Orgueil	Not found in any samples (noise).
68	0.0092	362	[9866.752, 9912.304]	[2.128, 1.824]	Atacama, Lignite	Unknown compound found in Lignite. Not found in Atacama (noise).
69	-0.0090	36	[2200.0, 2203.504]	[3.12, 2.2]	Atacama, Lignite, Aguas Zarcas, EET 96029	Not found in any samples (noise).
70	0.0084	220	[5010.208, 5013.712]	[2.872, 2.096]	Iceland	-
70	0.0084	220	[4645.792, 4645.792]	[2.784, 2.784]	Iceland	-
70	0.0084	220	[7112.608, 7112.608]	[2.624, 2.32]	Iceland	-
70	0.0084	220	[6884.848, 6884.848]	[2.672, 2.672]	Iceland	-
70	0.0084	220	[6394.288, 6394.288]	[3.376, 3.376]	Iceland	-
70	0.0084	220	[7547.104, 7547.104]	[3.344, 3.344]	Iceland	-
70	0.0084	220	[3097.024, 3097.024]	[3.08, 3.08]	Iceland	-
70	0.0084	112	[2655.52, 2655.52]	[3.272, 3.272]	Iceland	-
70	0.0084	112	[3885.424, 3885.424]	[3.08, 3.08]	Iceland	-
70	0.0084	112	[3780.304, 3780.304]	[2.976, 2.976]	Iceland	-
70	0.0084	112	[4449.568, 4449.568]	[3.128, 3.128]	Iceland	-
70	0.0084	220	[2855.248, 2855.248]	[3.16, 3.16]	Iceland	-
70	0.0084	467	[9547.888, 9586.432]	[1.088, 1.064]	Iceland	-
70	0.0084	464	[5388.64, 5388.64]	[2.92, 2.92]	Iceland	-
70	0.0084	220	[3167.104, 3167.104]	[2.344, 2.344]	Iceland	-
70	0.0084	220	[3521.008, 3521.008]	[3.08, 3.08]	Iceland	-
70	0.0084	220	[3717.232, 3717.232]	[2.984, 2.984]	Iceland	-
70	0.0084	220	[4519.648, 4519.648]	[3.456, 3.456]	Iceland	-
71	0.0083	467	[11324.416, 11355.952]	[1.088, 1.048]	Iceland, Utah, Aguas Zarcas, Jbilet Winselwan	Not found in any samples (noise).
72	-0.0082	464	[6464.368, 6513.424]	[2.408, 2.192]	Orgueil, Aguas Zarcas	Not found in any samples (noise).
73	0.0079	40	[9046.816, 9046.816]	[1.984, 1.984]	Atacama	-
73	0.0079	109	[8692.912, 8692.912]	[3.032, 3.032]	Atacama	-
73	0.0079	100	[8762.992, 8762.992]	[0.6, 0.6]	Atacama	-
73	0.0079	319	[10147.072, 10147.072]	[1.584, 1.584]	Atacama	-
74	-0.0077	111	[8934.688, 8934.688]	[0.64, 0.64]	Murchison	-
74	-0.0077	174	[9148.432, 9148.432]	[3.472, 3.472]	Murchison	-

Rank	Coef	m/z	RT1	RT2	Samples	Identified Compound
74	-0.0077	396	[8906.656, 8906.656]	[3.448, 3.448]	Murchison	-
75	-0.0074	214	[7018.0, 7067.056]	[2.792, 1.88]	Orgueil, Jbilet Winselwan, LEW 85311	Not found in any samples (noise).
75	-0.0074	132	[4582.72, 4607.248]	[1.208, 1.008]	LON 94101, LEW 85311	Not found in any samples (noise).
76	0.0069	220	[8307.472, 8310.976]	[3.296, 2.616]	Iceland, Orgueil	Not found in any samples (noise).
76	0.0069	220	[6947.92, 6982.96]	[2.744, 1.864]	Iceland, Orgueil	Not found in any samples (noise).
76	0.0069	464	[7596.16, 7641.712]	[2.232, 1.888]	Iceland, Green R. Shale, Orgueil, LEW 85311	Not found in any samples (noise).
77	-0.0067	503	[8976.736, 8976.736]	[2.464, 2.464]	Aguas Zarcas	-
77	-0.0067	464	[6215.584, 6215.584]	[2.488, 2.488]	Aguas Zarcas	-
77	-0.0067	464	[5696.992, 5707.504]	[2.768, 2.36]	Aguas Zarcas	-
77	-0.0067	503	[8903.152, 8903.152]	[2.464, 2.464]	Aguas Zarcas	-
77	-0.0067	132	[5570.848, 5605.888]	[3.344, 1.92]	Aguas Zarcas	-
77	-0.0067	464	[7876.48, 7876.48]	[2.84, 2.84]	Aguas Zarcas	-
77	-0.0067	464	[6601.024, 6601.024]	[3.2, 3.2]	Aguas Zarcas	-
77	-0.0067	453	[8801.536, 8833.072]	[1.088, 1.08]	Aguas Zarcas	-
77	-0.0067	464	[5637.424, 5637.424]	[3.448, 3.448]	Aguas Zarcas	-
77	-0.0067	464	[2361.184, 2361.184]	[3.016, 3.016]	Aguas Zarcas	-
77	-0.0067	464	[2441.776, 2441.776]	[2.176, 2.176]	Aguas Zarcas	-
77	-0.0067	94	[9015.28, 9015.28]	[1.144, 1.144]	Aguas Zarcas	-
77	-0.0067	124	[7978.096, 7978.096]	[1.24, 1.24]	Aguas Zarcas	-
77	-0.0067	464	[3909.952, 3909.952]	[2.072, 2.072]	Aguas Zarcas	-
77	-0.0067	464	[3391.36, 3391.36]	[2.24, 2.24]	Aguas Zarcas	-
78	-0.0066	172	[6996.976, 7025.008]	[1.112, 1.032]	Orgueil, ALH 83100, LEW 85311	Not found in any samples (noise).
79	-0.0061	132	[8398.576, 8398.576]	[1.952, 1.952]	LON 94101	-
79	-0.0061	132	[8142.784, 8177.824]	[3.0, 2.776]	LON 94101	-
80	0.0059	110	[3773.296, 3804.832]	[1.536, 1.504]	Rio Tinto	-
80	0.0059	174	[10353.808, 10353.808]	[2.808, 2.808]	Rio Tinto	-
81	-0.0054	171	[8437.12, 8468.656]	[1.112, 1.016]	Orgueil, ALH 83100	Sulfur background in Orgueil sample, not found in ALH 83100 sample (noise).
81	-0.0054	171	[8307.472, 8324.992]	[1.112, 1.008]	Orgueil, ALH 83100	Sulfur background in Orgueil sample, not found in ALH 83100 sample (noise).
82	0.0053	150	[8500.192, 8521.216]	[3.024, 2.744]	GSFC, LON 94101	Not found in any samples (noise).

Rank	Coef	m/z	RT1	RT2	Samples	Identified Compound
82	-0.0053	216	[7943.056, 7960.576]	[2.544, 1.92]	Lignite, Orgueil, ALH 83100, LEW 85311	Possible retene (formula C18H18) for Lignite and Orgueil samples, different unknown compound for LEW 85311 sample, not found in ALH 83100 sample (noise).
83	0.0051	100	[9323.632, 9323.632]	[0.944, 0.944]	Murchison Soil	-
84	-0.0047	214	[4747.408, 4789.456]	[3.248, 2.952]	Jbilet Winselwan	-
84	-0.0047	214	[3591.088, 3591.088]	[3.04, 3.04]	Jbilet Winselwan	-
84	-0.0047	214	[3216.16, 3216.16]	[1.264, 1.264]	Jbilet Winselwan	-
84	-0.0047	464	[4715.872, 4715.872]	[2.936, 2.936]	Jbilet Winselwan	-
84	-0.0047	214	[6050.896, 6057.904]	[3.184, 3.0]	Jbilet Winselwan	-
84	-0.0047	369	[11110.672, 11159.728]	[3.296, 3.272]	Jbilet Winselwan	-
84	-0.0047	563	[11187.76, 11187.76]	[2.664, 2.664]	Jbilet Winselwan	-
85	0.0046	467	[10945.984, 10988.032]	[1.088, 1.064]	Utah, Aguas Zarcas	Not found in any samples (noise).
85	0.0046	467	[10826.848, 10861.888]	[1.08, 1.048]	Utah, Aguas Zarcas	Not found in any samples (noise).
85	0.0046	464	[5760.064, 5795.104]	[3.328, 2.648]	Utah, Aguas Zarcas	Not found in any samples (noise).
86	-0.0041	242	[6615.04, 6653.584]	[1.088, 1.04]	Green R. Shale, Orgueil, ALH 83100	Unknown compounds in Green R. Shale. The rest are noise.
87	-0.0032	464	[8363.536, 8367.04]	[2.96, 2.944]	Iceland, EET 96029	Not found in any samples (noise).
88	-0.0030	467	[10402.864, 10441.408]	[1.08, 1.048]	Iceland, Aguas Zarcas, Jbilet Winselwan	Not found in any samples (noise).
89	-0.0027	230	[7771.36, 7778.368]	[2.2, 1.944]	Orgueil, LEW 85311	Two different compounds in the two samples, both with a formula of C18H14.
90	-0.0026	144	[8009.632, 8055.184]	[1.04, 1.024]	Green R. Shale, ALH 83100	Not found in any samples (noise).
90	0.0026	304	[9218.512, 9246.544]	[2.824, 2.424]	Green R. Shale, Lignite	Different unknown compounds in both samples.
90	0.0026	227	[8759.488, 8759.488]	[1.728, 1.536]	Green R. Shale, Lignite	Different unknown compounds in both samples.
91	0.0017	467	[10711.216, 10725.232]	[1.088, 1.072]	Iceland, Aguas Zarcas	Not found in any samples (noise).
92	-0.0015	134	[7273.792, 7301.824]	[1.232, 1.216]	Orgueil	-
92	-0.0015	192	[7491.04, 7526.08]	[1.44, 1.4]	Orgueil	-
92	-0.0015	31	[4572.208, 4614.256]	[2.936, 2.368]	Orgueil	-
92	-0.0015	31	[4162.24, 4190.272]	[3.336, 2.64]	Orgueil	-
92	-0.0015	31	[4102.672, 4102.672]	[2.808, 2.808]	Orgueil	-
92	-0.0015	31	[4046.608, 4046.608]	[3.232, 3.232]	Orgueil	-
92	-0.0015	31	[2897.296, 2946.352]	[3.16, 1.832]	Orgueil	-
92	-0.0015	31	[4249.84, 4249.84]	[2.952, 2.952]	Orgueil	-

Rank	Coef	m/z	RT1	RT2	Samples	Identified Compound
92	-0.0015	31	[3447.424, 3489.472]	[2.976, 1.952]	Orgueil	-
92	-0.0015	31	[3356.32, 3405.376]	[3.464, 2.904]	Orgueil	-
92	-0.0015	31	[4502.128, 4512.64]	[2.568, 2.288]	Orgueil	-
92	-0.0015	31	[3079.504, 3128.56]	[2.664, 1.328]	Orgueil	-
92	-0.0015	31	[2904.304, 2918.32]	[1.464, 1.032]	Orgueil	-
92	-0.0015	64	[7378.912, 7403.44]	[2.472, 2.36]	Orgueil	-
92	-0.0015	369	[9540.88, 9575.92]	[1.96, 1.96]	Orgueil	-
92	-0.0015	464	[7203.712, 7203.712]	[3.36, 3.36]	Orgueil	-
92	-0.0015	464	[7256.272, 7277.296]	[3.456, 2.92]	Orgueil	-
92	-0.0015	214	[8023.648, 8023.648]	[3.296, 3.296]	Orgueil	-
92	-0.0015	214	[7939.552, 7939.552]	[3.4, 3.4]	Orgueil	-
93	0.0013	142	[9726.592, 9772.144]	[2.416, 2.0]	Lignite	-
93	0.0013	54	[11345.44, 11345.44]	[2.432, 2.432]	Lignite	-
93	0.0013	153	[9421.744, 9421.744]	[1.976, 1.976]	Lignite	-
93	0.0013	436	[11422.528, 11422.528]	[2.424, 2.424]	Lignite	-
93	0.0013	471	[10700.704, 10700.704]	[2.064, 2.064]	Lignite	-
93	0.0013	83	[10175.104, 10175.104]	[2.624, 2.624]	Lignite	-
93	0.0013	290	[9463.792, 9463.792]	[0.04, 0.04]	Lignite	-
93	0.0013	289	[9463.792, 9463.792]	[3.408, 3.408]	Lignite	-
93	0.0013	305	[9155.44, 9155.44]	[2.128, 2.128]	Lignite	-
93	0.0013	271	[10290.736, 10290.736]	[0.136, 0.136]	Lignite	-
93	0.0013	275	[8454.64, 8454.64]	[1.992, 1.992]	Lignite	-
93	0.0013	394	[10595.584, 10595.584]	[2.208, 2.208]	Lignite	-
93	0.0013	420	[9810.688, 9810.688]	[2.528, 2.528]	Lignite	-
93	0.0013	310	[10893.424, 10893.424]	[2.28, 2.28]	Lignite	-
93	0.0013	321	[10753.264, 10753.264]	[3.384, 3.384]	Lignite	-
93	0.0013	360	[9968.368, 9968.368]	[2.592, 2.592]	Lignite	-
94	0.0012	294	[9341.152, 9341.152]	[1.984, 1.984]	Green R. Shale	-
94	0.0012	304	[9358.672, 9358.672]	[2.912, 2.912]	Green R. Shale	-
94	0.0012	288	[9299.104, 9299.104]	[3.184, 3.184]	Green R. Shale	-

Rank	Coef	m/z	RT1	RT2	Samples	Identified Compound
94	0.0012	369	[10507.984, 10507.984]	[2.72, 2.72]	Green R. Shale	-
94	0.0012	228	[8226.88, 8226.88]	[3.416, 3.416]	Green R. Shale	-
94	0.0012	228	[8230.384, 8230.384]	[0.048, 0.048]	Green R. Shale	-

Table S6. Filtering Noisy Regions. Regions with intensities exceeding half of the maximum were analyzed by setting intensities below this threshold to zero (see Fig. S8). A ratio test was conducted to assess the proportion of non-zero pixels within these regions. Peaks were excluded if this ratio surpassed the specific thresholds listed, which details the defined regions and their respective non-zero pixel ratio thresholds

RT1 region (min)	RT2 region (s)	Threshold
[30, 190]	[0, 1]	10^{-3}
[145, 190]	[1.1, 1.8]	10^{-2}
[144.8, 145.1]	[2.2, 3]	10^{-2}
[85.4 , 87.0]	[0, 3.5]	10^{-1}
[87.5 , 89.1]	[0, 1.8]	10^{-2}
[127.5, 129.16]	[0, 3.5]	10^{-1}
[144.16, 145.8]	[0, 3.5]	10^{-2}

References

1. H Naraoka, , et al., Soluble organic molecules in samples of the carbonaceous asteroid (162173) ryugu. *Science* **379** (2023).
2. P Schmitt-Kopplin, N Hertkorn, M Harir, , et al., Soluble organic matter molecular atlas of ryugu reveals cold hydrothermalism on c-type asteroid parent body. *Nat. Commun*. **14**, 6525 (2023).
3. JC Aponte, , et al., Pahs, hydrocarbons, and dimethylsulfides in asteroid ryugu samples a0106 and c0107 and the orgueil (ci1) meteorite. *Earth, Planets Space* **75**, 28 (2023).
4. J Aponte, D Whitaker, M Powner, J Elsila, J Dworkin, Analyses of aliphatic aldehydes and ketones in carbonaceous chondrites. *ACS Earth Space Chem*. **3**, 463–472 (2019).
5. D Fernández-Remolar, et al., The tinto river, an extreme acidic environment under control of iron, as an analog of the terra meridiani hematite site of mars. *Planet. Space Sci*. **52**, 239–248 (2004) Exploring Mars Surface and its Earth Analogues.
6. R Amils, D Fernández-Remolar, TI Team, Río tinto: A geochemical and mineralogical terrestrial analogue of mars. *Life* **4**, 511–534 (2014).
7. NW Hinman, et al., Targeting mixtures of jarosite and clay minerals for Mars exploration. *Am. Mineral*. **106**, 1237–1254 (2021).
8. KM Seaton, CI Pozarycki, N Nuñez, AMa Stockton, A robust capillary electrophoresis with laser-induced fluorescence detection (ce-lif) method for quantitative compositional analysis of trace amino acids in hypersaline samples. *ACS Earth Space Chem*. **7**, 2214–2221 (2023).
